# Supplementary material for: Cross-Country Adaptation of a Psychological Flexibility Measure: The Comprehensive Assessment of Acceptance and Commitment Therapy Processes
Source: Int J Environ Res Public Health. 2022 Mar 8;19(6):3150. doi: 10.3390/ijerph19063150 (PMC8953951; doi:10.3390/ijerph19063150)
Supplement: Supplementary file 1 [file ijerph-19-03150-s001.zip › Supplementary File 1 - Italian CompACT Translation Grid.pdf]

Article title: Cross-country adaptation of a psychological flexibility measure: The Comprehensive assessment of Acceptance and Commitment Therapy processes

Authors: Ambra Mara Giovannetti, Jana Pöttgen, Elisenda Anglada, Rebeca Menendez, Jürgen Hoyer, Andrea Giordano, Kenneth Ian Pakenham, Ingrid Galán, Alessandra Solari

Corresponding author: Ambra Mara Giovannetti, ambra.giovannetti@istituto-besta.it
Fondazione IRCCS Istituto Neurologico Carlo Besta, Milan, Italy. Via Celoria 11, 20133 Milano, Italia

Supplementary File 1 “Italian CompACT Translation grid”

COMMENTS/QUESTIONS MADE BY:
PP (Consultant Italian translator 1)<br>FB (Consultant Italian translator 2)<br>LR (Consultant backward translator)<br><br>AS (Researcher 1)<br>AMG (Researcher2)<br>MAF (Lay person)<br>NP (ACT expert)<br>AO (ACT expert)<br><br>☞ Notes/queries for the scale authors<br>*Author: Nima Moghaddam*<br>*Author: Dave Dawson*

TITLE

ORIGINAL ENGLISH WORDING	COMMENTS/QUESTIONS
CompACT (Comprehensive assessment of Acceptance and Commitment Therapy processes)	
**ITALIAN TRANSLATION 1**	
CompACT (Valutazione generale dei processi di terapia di accettazione e impegno)	
**ITALIAN TRANSLATION 2**	
CompACT /Valutazione complessiva del processo per la terapia di accettazione e di impegno	
**RECONCILED ITALIAN TRANSLATION**	
CompACT (valutazione globale dei processi della Terapia di Accettazione e Impegno nell'azione)	
**BACKWARD TRANSLATION**	
CompACT (Global Assessment Of Acceptance And Commitment Therapy Processes In Action)	
**ADVANCED ITALIAN VERSION 1**	As for other PROMs, it was decided not to translate the title
CompACT (Comprehensive assessment of Acceptance and Commitment Therapy	

processes)	
**ADVANCED ITALIAN VERSION 2 (post discussion with ACT expert)**	
CompACT (Comprehensive assessment of Acceptance and Commitment Therapy processes)	
**FINAL ITALIAN TRANSLATION**	
CompACT (Comprehensive assessment of Acceptance and Commitment Therapy processes)	

INTRODUCTION

ORIGINAL ENGLISH WORDING	COMMENTS/QUESTIONS
Please rate the following 23 statements using the scale below:	
**ITALIAN TRANSLATION 1**	
Assegnate un punteggio a ciascuna delle 23 affermazioni che seguono, in base a questa scala:	
**ITALIAN TRANSLATION 2**	
Per favore valuta le seguenti 23 affermazioni secondo la scala qui riportata	
**RECONCILED ITALIAN TRANSLATION**	Note: the original statement was rephrased to improve clarity.
Per ciascuna delle 23 affermazioni che seguono, indica con una crocetta il tuo grado di accordo/disaccordo	
**BACKWARD TRANSLATION**	1☞ To be checked by the scale author (is it OK?)
Check the box that best matches how much you agree or disagree with each of the following 23 statements.	*The wording seems fine, assuming that the form is presented with check-boxes for each of the seven response options. Does depend on the presentation format though (for example, in the original template form, we had a numbered scale for which respondents would circle [paper version] or select [electronic version] their responses) but the wording could presumably be adapted if needs be (e.g., 'select the option…' versus 'check the box…')*
**ADVANCED ITALIAN VERSION 1**	Reconciled OK. No comments from the ACT expert.
Per ciascuna delle 23 affermazioni che seguono, indichi con una crocetta il suo grado di accordo/disaccordo	
**ADVANCED ITALIAN VERSION 2 (post discussion with ACT expert)**	
Per ciascuna delle 23 affermazioni che seguono, indichi con una crocetta il suo grado di accordo/disaccordo	
**FINAL ITALIAN TRANSLATION**	

Per ciascuna delle 23 affermazioni che seguono, indichi con una crocetta il suo grado di accordo/disaccordo	

POSSIBLE REPLY # 1

ORIGINAL ENGLISH WORDING	COMMENTS/QUESTIONS
Strongly disagree	
**ITALIAN TRANSLATION 1**	
È decisamente falso	
**ITALIAN TRANSLATION 2**	
Fortemente in disaccordo	
**RECONCILED ITALIAN TRANSLATION**	
Totalmente in disaccordo	
**BACKWARD TRANSLATION**	
Disagree totally	
**ADVANCED ITALIAN VERSION 1**	Reconciled OK. No comments from the ACT expert.
Totalmente in disaccordo	
**ADVANCED ITALIAN VERSION 2 (post discussion with ACT expert)**	
Totalmente in disaccordo	
**FINAL ITALIAN TRANSLATION**	
Totalmente in disaccordo	

POSSIBLE REPLY # 2

ORIGINAL ENGLISH WORDING	COMMENTS/QUESTIONS
Moderately disagree	
**ITALIAN TRANSLATION 1**	
Non è molto vero	
**ITALIAN TRANSLATION 2**	
Moderatamente in disaccordo	
**RECONCILED ITALIAN TRANSLATION**	
Moderatamente in disaccordo	
**BACKWARD TRANSLATION**	
Disagree moderately	
**ADVANCED ITALIAN VERSION 1**	Reconciled OK. No comments from the ACT expert.
Moderatamente in disaccordo	

ADVANCED ITALIAN VERSION 2 (post discussion with ACT expert)	
Moderatamente in disaccordo	
**FINAL ITALIAN TRANSLATION**	
Moderatamente in disaccordo	

POSSIBLE REPLY # 3

ORIGINAL ENGLISH WORDING	COMMENTS/QUESTIONS
Slightly disagree	
**ITALIAN TRANSLATION 1**	
Non è del tutto vero	
**ITALIAN TRANSLATION 2**	
Leggermente in disaccordo	
**RECONCILED ITALIAN TRANSLATION**	
Leggermente in disaccordo	
**BACKWARD TRANSLATION**	
Disagree slightly	
**ADVANCED ITALIAN VERSION 1**	Reconciled OK. No comments from the ACT expert.
Leggermente in disaccordo	
**ADVANCED ITALIAN VERSION 2 (post discussion with ACT expert)**	
Leggermente in disaccordo	
**FINAL ITALIAN TRANSLATION**	
Leggermente in disaccordo	

POSSIBLE REPLY # 4

ORIGINAL ENGLISH WORDING	COMMENTS/QUESTIONS
Neither agree nor disagree	
**ITALIAN TRANSLATION 1**	
Non è né vero né falso	
**ITALIAN TRANSLATION 2**	
Nè d'accordo né in disaccordo	
**RECONCILED ITALIAN TRANSLATION**	
Nè d'accordo né in disaccordo	
**BACKWARD TRANSLATION**	
Neither agree nor disagree	

**ADVANCED ITALIAN VERSION 1**	Reconciled OK. No comments from the ACT expert.
Nè d'accordo né in disaccordo	
**ADVANCED ITALIAN VERSION 2 (post discussion with ACT expert)**	
Nè d'accordo né in disaccordo	
**FINAL ITALIAN TRANSLATION**	
Nè d'accordo né in disaccordo	

POSSIBLE REPLY # 5

**ORIGINAL ENGLISH WORDING**	**COMMENTS/QUESTIONS**
Slightly agree	
**ITALIAN TRANSLATION 1**	
Non è del tutto falso	
**ITALIAN TRANSLATION 2**	
Leggermente d'accordo	
**RECONCILED ITALIAN TRANSLATION**	
Leggermente d'accordo	
**BACKWARD TRANSLATION**	
Agree slightly	
**ADVANCED ITALIAN VERSION 1**	Reconciled OK. No comments from the ACT expert.
Leggermente d'accordo	
**ADVANCED ITALIAN VERSION 2 (post discussion with ACT expert)**	
Leggermente d'accordo	
**FINAL ITALIAN TRANSLATION**	
Leggermente d'accordo	

POSSIBLE REPLY # 6

**ORIGINAL ENGLISH WORDING**	**COMMENTS/QUESTIONS**
Moderately agree	
**ITALIAN TRANSLATION 1**	
È abbastanza vero	
**ITALIAN TRANSLATION 2**	
Moderatamente d'accordo	
**RECONCILED ITALIAN TRANSLATION**	
Moderatamente d'accordo	

BACKWARD TRANSLATION	
Agree moderately	
ADVANCED ITALIAN VERSION 1	Reconciled OK. No comments from the ACT expert.
Moderatamente d'accordo	
ADVANCED ITALIAN VERSION 2 (post discussion with ACT expert)	
Moderatamente d'accordo	
FINAL ITALIAN TRANSLATION	
Moderatamente d'accordo	

POSSIBLE REPLY # 7

ORIGINAL ENGLISH WORDING	COMMENTS/QUESTIONS
Strongly agree	
ITALIAN TRANSLATION 1	
È decisamente vero	
ITALIAN TRANSLATION 2	
Fortemente d'accordo	
RECONCILED ITALIAN TRANSLATION	
Totalmente d'accordo	
BACKWARD TRANSLATION	
Agree Totally	
ADVANCED ITALIAN VERSION 1	Reconciled OK. No comments from the ACT expert.
Totalmente d'accordo	
ADVANCED ITALIAN VERSION 2 (post discussion with ACT expert)	
Totalmente d'accordo	
FINAL ITALIAN TRANSLATION	
Totalmente d'accordo	

ITEM # 1

ORIGINAL ENGLISH WORDING	COMMENTS/QUESTIONS
I can identify the things that really matter to me in life and pursue them	
ITALIAN TRANSLATION 1	
Sono in grado di capire quali cose sono veramente importanti per me nella vita, e di perseguirle	
ITALIAN TRANSLATION 2	

Sono in grado di riconoscere ciò che nella vita è veramente importante per me e di impegnarmi per ottenerlo	
**RECONCILED ITALIAN TRANSLATION**	
Sono in grado di capire quali cose sono veramente importanti per me nella vita, e di perseguirle	
**BACKWARD TRANSLATION**	
I am able to understand the things that really matter to me in my life, and to pursue them	
**ADVANCED ITALIAN VERSION 1**	Reconciled version revised OK<br>I suggest to change "perseguirle" with "portarle avanti".
Sono in grado di riconoscere le cose che sono veramente importanti per me nella vita, e di perseguirle	
**ADVANCED ITALIAN VERSION 2 (post discussion with ACT expert)**	
Sono in grado di riconoscere le cose che sono veramente importanti per me nella vita e di portarle avanti	
**FINAL ITALIAN TRANSLATION**	
Sono in grado di riconoscere le cose che sono veramente importanti per me nella vita e di portarle avanti	

ITEM # 2

ORIGINAL ENGLISH WORDING	COMMENTS/QUESTIONS
One of my big goals is to be free from painful emotions	
**ITALIAN TRANSLATION 1**	
Una delle mie aspirazioni è essere libero da emozioni dolorose	
**ITALIAN TRANSLATION 2**	
Uno dei miei obiettivi principali è quello di essere libero dalle sofferenze	
**RECONCILED ITALIAN TRANSLATION**	
Uno dei miei obiettivi principali è quello di essere libero dalle emozioni dolorose	
**BACKWARD TRANSLATION**	
One of my main goals is to be free of painful emotions	
**ADVANCED ITALIAN VERSION 1**	Reconciled OK<br>"essere libero" is wrong because it is passive. To be coherent with the ACT theory, this should be transformed in the active form, such as "liberarmi da…" or "non avere emozioni…"
Uno dei miei obiettivi principali è quello di essere libero dalle emozioni dolorose	
**ADVANCED ITALIAN VERSION 2 (post discussion with ACT expert)**	
Uno dei miei obiettivi principali è quello di non avere emozioni dolorose	
**FINAL ITALIAN TRANSLATION**	

Uno dei miei obiettivi principali è quello di non avere emozioni dolorose	

ITEM # 3

**ORIGINAL ENGLISH WORDING**	**COMMENTS/QUESTIONS**
I rush through meaningful activities without being really attentive to them	
**ITALIAN TRANSLATION 1**	
Eseguo in gran fretta anche le cose importanti, senza in realtà dedicare loro attenzione	
**ITALIAN TRANSLATION 2**	
Faccio tante cose senza prestarci troppa attenzione	
**RECONCILED ITALIAN TRANSLATION**	
Svolgo frettolosamente anche attività importanti, senza in realtà dedicare loro attenzione	
**BACKWARD TRANSLATION**	
I rush through things including important activities, without actually paying attention to them	
**ADVANCED ITALIAN VERSION 1**	Reconciled revised: “anche” removed
Svolgo frettolosamente attività importanti, senza in realtà dedicare loro attenzione	
**ADVANCED ITALIAN VERSION 2 (post discussion with ACT expert)**	I suggest to change “dedicare” with “perseguire”
Svolgo frettolosamente attività importanti, senza in realtà prestare loro attenzione	
**FINAL ITALIAN TRANSLATION**	
Svolgo frettolosamente attività importanti, senza in realtà prestare loro attenzione	

ITEM # 4

**ORIGINAL ENGLISH WORDING**	**COMMENTS/QUESTIONS**
I try to stay busy to keep thoughts or feelings from coming	
**ITALIAN TRANSLATION 1**	
Cerco di tenermi occupato per non pensare o non farmi prendere dai sentimenti	
**ITALIAN TRANSLATION 2**	
Cerco di tenermi occupato per evitare di pensare o di provare emozioni	
**RECONCILED ITALIAN TRANSLATION**	
Cerco di tenermi occupato per evitare pensieri o emozioni	
**BACKWARD TRANSLATION**	
I try to keep myself busy to avoid thoughts or emotions	
**ADVANCED ITALIAN VERSION 1**	Reconciled OK. No comments from the ACT expert.
Cerco di tenermi occupato per evitare pensieri o emozioni	

**ADVANCED ITALIAN VERSION 2 (post discussion with ACT expert)**	
Cerco di tenermi occupato per evitare pensieri o emozioni	
**FINAL ITALIAN TRANSLATION**	
Cerco di tenermi occupato per evitare pensieri o emozioni	

ITEM # 5

ORIGINAL ENGLISH WORDING	COMMENTS/QUESTIONS
I act in ways that are consistent with how I wish to live my life	
**ITALIAN TRANSLATION 1**	
Mi comporto in maniera coerente con il modo in cui desidero vivere la vita	
**ITALIAN TRANSLATION 2**	
Agisco in modo coerente a come vorrei vivere la mia vita	
**RECONCILED ITALIAN TRANSLATION**	
Agisco in modo coerente a come desidero vivere la mia vita	
**BACKWARD TRANSLATION**	
I act consistently with the way I want to live my life	
**ADVANCED ITALIAN VERSION 1**	Reconciled OK. No comments from the ACT expert.
Agisco in modo coerente a come desidero vivere la mia vita	
**ADVANCED ITALIAN VERSION 2 (post discussion with ACT expert)**	
Agisco in modo coerente a come desidero vivere la mia vita	
**FINAL ITALIAN TRANSLATION**	
Agisco in modo coerente a come desidero vivere la mia vita	

ITEM # 6

ORIGINAL ENGLISH WORDING	COMMENTS/QUESTIONS
I get so caught up in my thoughts that I am unable to do the things that I most want to do	
**ITALIAN TRANSLATION 1**	
Sono talmente preso nei miei pensieri che non riesco a fare le cose che più voglio fare	
**ITALIAN TRANSLATION 2**	
Mi faccio così tanto coinvolgere dai miei pensieri da non essere in grado di fare ciò che più voglio fare	
**RECONCILED ITALIAN TRANSLATION**	
Sono talmente preso nei miei pensieri da non riuscire a fare le cose che più voglio fare	
**BACKWARD TRANSLATION**	

I am so taken up by my thoughts that I am unable to do the things I really want to do	
**ADVANCED ITALIAN VERSION 1**	Reconciled OK. No comments from the ACT expert.
Sono talmente preso nei miei pensieri da non riuscire a fare le cose che più voglio fare	
**ADVANCED ITALIAN VERSION 2 (post discussion with ACT expert)**	
Sono talmente preso nei miei pensieri da non riuscire a fare le cose che più voglio fare	
**FINAL ITALIAN TRANSLATION**	
Sono talmente preso nei miei pensieri da non riuscire a fare le cose che più voglio fare	

ITEM # 7

ORIGINAL ENGLISH WORDING	COMMENTS/QUESTIONS
I make choices based on what is important to me, even if it is stressful	
**ITALIAN TRANSLATION 1**	
Faccio le mie scelte in base a quello che per me è importante, anche quando mi costa fatica	
**ITALIAN TRANSLATION 2**	
Faccio scelte basate su quello che per me è importante, anche se questo è stressante	
**RECONCILED ITALIAN TRANSLATION**	
Faccio scelte in base a quello che per me è importante, anche quando mi costa fatica	
**BACKWARD TRANSLATION**	**2**☞ To be checked by the scale author (is “takes effort” acceptable instead of “stressful” in this context? In Italian “stressante” is a rather general term that the panel preferred to avoid, but feedback from the author is required)<br>*Yes - this seems to capture the gist of the item (being able to select actions that are values-consistent, even when it may be difficult or demanding to do so)*<br>*I agree; I wasn’t sure about ‘takes effort’ as that doesn’t seem to capture the aversive nature of the phenomenon. However, ‘fatica’ appears related to fatigue, exertion, etc. so I think that is fine.*
I make decisions based on what matters to me, even when it takes effort	
**ADVANCED ITALIAN VERSION 1**	Reconciled OK. No comments from the ACT expert.
Faccio scelte in base a quello che per me è importante, anche quando mi costa fatica	
**ADVANCED ITALIAN VERSION 2 (post discussion with ACT expert)**	
Faccio scelte in base a quello che per me è importante, anche quando mi costa fatica	
**FINAL ITALIAN TRANSLATION**	
Faccio scelte in base a quello che per me è importante, anche quando mi costa fatica	

ITEM # 8

ORIGINAL ENGLISH WORDING	COMMENTS/QUESTIONS
I tell myself that I shouldn't have certain thoughts	
**ITALIAN TRANSLATION 1**	
Dico a me stesso che non dovrei pensare a certe cose	
**ITALIAN TRANSLATION 2**	
Mi dico che non dovrei avere certi pensieri	
**RECONCILED ITALIAN TRANSLATION**	
Dico a me stesso che non dovrei avere certi pensieri	
**BACKWARD TRANSLATION**	
I tell myself I should not have certain thoughts	
**ADVANCED ITALIAN VERSION 1**	Reconciled OK. No comments from the ACT expert.
Dico a me stesso che non dovrei avere certi pensieri	
**ADVANCED ITALIAN VERSION 2 (post discussion with ACT expert)**	
Dico a me stesso che non dovrei avere certi pensieri	
**FINAL ITALIAN TRANSLATION**	
Dico a me stesso che non dovrei avere certi pensieri	

ITEM # 9

ORIGINAL ENGLISH WORDING	COMMENTS/QUESTIONS
I find it difficult to stay focused on what's happening in the present	
**ITALIAN TRANSLATION 1**	
Faccio fatica a rimanere concentrato su quello che sta accadendo nel presente	
**ITALIAN TRANSLATION 2**	
Trovo difficile rimanere concentrato su quello che succede al momento	
**RECONCILED ITALIAN TRANSLATION**	
Faccio fatica a rimanere concentrato su ciò che accade nel presente	
**BACKWARD TRANSLATION**	
I struggle to stay focussed on what is happening right now	
**ADVANCED ITALIAN VERSION 1**	Reconciled OK. No comments from the ACT expert.
Faccio fatica a rimanere concentrato su ciò che accade nel presente	
**ADVANCED ITALIAN VERSION 2 (post discussion with ACT expert)**	
Faccio fatica a rimanere concentrato su ciò che accade nel presente	
**FINAL ITALIAN TRANSLATION**	

Faccio fatica a rimanere concentrato su ciò che accade nel presente	

ITEM # 10

ORIGINAL ENGLISH WORDING	COMMENTS/QUESTIONS
I behave in line with my personal values	
**ITALIAN TRANSLATION 1**	
Mi comporto secondo i miei valori personali	
**ITALIAN TRANSLATION 2**	
Mi comporto secondo i miei valori personali	
**RECONCILED ITALIAN TRANSLATION**	
Mi comporto secondo i miei valori personali	
**BACKWARD TRANSLATION**	"Personal beliefs" probably not equivalent to "values". However, the Italian reconciled is OK "values"="valori"
I behave according to my personal beliefs	
**ADVANCED ITALIAN VERSION 1**	Reconciled OK. No comments from the ACT expert.
Mi comporto secondo i miei valori personali	
**ADVANCED ITALIAN VERSION 2 (post discussion with ACT expert)**	
Mi comporto secondo i miei valori personali	
**FINAL ITALIAN TRANSLATION**	
Mi comporto secondo i miei valori personali	

ITEM # 11

ORIGINAL ENGLISH WORDING	COMMENTS/QUESTIONS
I go out of my way to avoid situations that might bring difficult thoughts, feelings, or sensations	
**ITALIAN TRANSLATION 1**	
Faccio di tutto per evitare situazioni che mi possano dare pensieri, sentimenti o sensazioni difficili da sopportare	
**ITALIAN TRANSLATION 2**	
Faccio di tutto per evitare situazioni che potrebbero generare pensieri , emozioni o sensazioni difficili	
**RECONCILED ITALIAN TRANSLATION**	
Faccio di tutto per evitare situazioni che potrebbero suscitare pensieri, emozioni o sensazioni spiacevoli	
**BACKWARD TRANSLATION**	
I do everything I can to avoid situations that might arouse unpleasant thoughts, emotions or	

feelings	
**ADVANCED ITALIAN VERSION 1**	Reconciled OK. No comments from the ACT expert.
Faccio di tutto per evitare situazioni che potrebbero suscitare pensieri, emozioni o sensazioni spiacevoli	
**ADVANCED ITALIAN VERSION 2 (post discussion with ACT expert)**	
Faccio di tutto per evitare situazioni che potrebbero suscitare pensieri, emozioni o sensazioni spiacevoli	
**FINAL ITALIAN TRANSLATION**	
Faccio di tutto per evitare situazioni che potrebbero suscitare pensieri, emozioni o sensazioni spiacevoli	

ITEM # 12

ORIGINAL ENGLISH WORDING	COMMENTS/QUESTIONS
Even when doing the things that matter to me, I find myself doing them without paying attention	
**ITALIAN TRANSLATION 1**	
Anche mentre faccio cose che per me sono importanti, mi accorgo che le faccio senza prestare attenzione	
**ITALIAN TRANSLATION 2**	
Anche quando faccio cose che per me sono importanti, mi trovo a farle senza prestare attenzione	
**RECONCILED ITALIAN TRANSLATION**	
Anche quando faccio cose che per me sono importanti, mi accorgo di farle senza prestare attenzione	
**BACKWARD TRANSLATION**	
Even when I do things that matter to me, I realise I'm doing them without paying attention	
**ADVANCED ITALIAN VERSION 1**	Reconciled OK
Anche quando faccio cose che per me sono importanti, mi accorgo di farle senza prestare attenzione	
**ADVANCED ITALIAN VERSION 2 (post discussion with ACT expert)**	
Anche quando faccio cose che per me sono importanti, mi accorgo di farle senza prestare attenzione	
**FINAL ITALIAN TRANSLATION**	
Anche quando faccio cose che per me sono importanti, mi accorgo di farle senza prestare attenzione	

ITEM # 13

ORIGINAL ENGLISH WORDING	COMMENTS/QUESTIONS
I am willing to fully experience whatever thoughts, feelings and sensations come up for me, without trying to change or defend against them	
**ITALIAN TRANSLATION 1**	
Sono disposto ad accettare l'esperienza di qualsiasi pensiero, sentimento e sensazione che mi si presenti, senza cercare di cambiarlo o di difendermene.	
**ITALIAN TRANSLATION 2**	
Sono disposto a vivere pienamente qualsiasi pensiero, emozione e sensazione mi si presenti senza cercare di cambiare o di proteggermi da queste	
**RECONCILED ITALIAN TRANSLATION**	I don't like 'difendermene', even if it is grammatically correct
Sono disposto a vivere pienamente qualsiasi pensiero, emozione e sensazione mi si presenti senza cercare di cambiarli o difendermene	
**BACKWARD TRANSLATION**	
I am willing to fully experience whatever thoughts, emotions or feelings come to me without trying to change them or defend myself from them	
**ADVANCED ITALIAN VERSION 1**	Reconciled OK. No comments from the ACT expert.
Sono disposto a vivere pienamente qualsiasi pensiero, emozione e sensazione mi si presenti senza cercare di cambiarli o difendermene	
**ADVANCED ITALIAN VERSION 2 (post discussion with ACT expert)**	
Sono disposto a vivere pienamente qualsiasi pensiero, emozione e sensazione mi si presenti senza cercare di cambiarli o difendermene	
**FINAL ITALIAN TRANSLATION**	
Sono disposto a vivere pienamente qualsiasi pensiero, emozione e sensazione mi si presenti senza cercare di cambiarli o difendermene	

ITEM # 14

ORIGINAL ENGLISH WORDING	COMMENTS/QUESTIONS
I undertake things that are meaningful to me, even when I find it hard to do so	
**ITALIAN TRANSLATION 1**	
Mi impegno a fare cose che per me sono importanti, anche quando trovo difficile farlo.	
**ITALIAN TRANSLATION 2**	
Mi impegno per cose che sono importanti per me, anche quando mi è difficile farlo	
**RECONCILED ITALIAN TRANSLATION**	

Mi impegno a fare cose che per me sono importanti, anche quando lo trovo difficile	
**BACKWARD TRANSLATION**	
I strive to do things that matter to me, even when I find it difficult	
**ADVANCED ITALIAN VERSION 1**	Reconciled OK. No comments from the ACT expert.
Mi impegno a fare cose che per me sono importanti, anche quando lo trovo difficile	
**ADVANCED ITALIAN VERSION 2 (post discussion with ACT expert)**	
Mi impegno a fare cose che per me sono importanti, anche quando lo trovo difficile	
**FINAL ITALIAN TRANSLATION**	
Mi impegno a fare cose che per me sono importanti, anche quando lo trovo difficile	

ITEM # 15

ORIGINAL ENGLISH WORDING	COMMENTS/QUESTIONS
I work hard to keep out upsetting feelings	
**ITALIAN TRANSLATION 1**	
Mi impegno molto a tenere lontani da me i sentimenti che mi fanno star male	
**ITALIAN TRANSLATION 2**	
Faccio di tutto per tenere lontano sentimenti negativi	
**RECONCILED ITALIAN TRANSLATION**	
Faccio di tutto per tenere lontano i sentimenti che mi fanno star male	
**BACKWARD TRANSLATION**	*With some of these, the back-translated wording seems more eloquent than the original!*<br>*I agree! This is much more nicely phrased than the original.*
I do everything I can to ward off feelings that upset me	
**ADVANCED ITALIAN VERSION 1**	Reconciled OK<br>I suggest to change "sentimenti" with "emozioni".
Faccio di tutto per tenere lontano i sentimenti che mi fanno star male	
**ADVANCED ITALIAN VERSION 2 (post discussion with ACT expert)**	
Faccio di tutto per tenere lontano le emozioni che mi fanno star male	
**FINAL ITALIAN TRANSLATION**	
Faccio di tutto per tenere lontano le emozioni che mi fanno star male	

ITEM # 16

ORIGINAL ENGLISH WORDING	COMMENTS/QUESTIONS
I do jobs or tasks automatically, without being aware of what I'm doing	
**ITALIAN TRANSLATION 1**	
Eseguo il lavoro o le cose che devo fare in modo automatico, senza rendermi conto	

di quello che sto facendo	
**ITALIAN TRANSLATION 2**	
Eseguo compiti o azioni in modo automatico, senza rendermi conto di quello che sto facendo	
**RECONCILED ITALIAN TRANSLATION**	
Eseguo compiti o mansioni in modo automatico, senza rendermi conto di quello che sto facendo	
**BACKWARD TRANSLATION**	
I perform tasks or jobs automatically, without being aware of what I am doing	
**ADVANCED ITALIAN VERSION 1**	Reconciled OK. No comments from the ACT expert.
Eseguo compiti o mansioni in modo automatico, senza rendermi conto di quello che sto facendo	
**ADVANCED ITALIAN VERSION 2 (post discussion with ACT expert)**	
Eseguo compiti o mansioni in modo automatico, senza rendermi conto di quello che sto facendo	
**FINAL ITALIAN TRANSLATION**	
Eseguo compiti o mansioni in modo automatico, senza rendermi conto di quello che sto facendo	

ITEM # 17

**ORIGINAL ENGLISH WORDING**	**COMMENTS/QUESTIONS**
I am able to follow my long terms plans including times when progress is slow	
**ITALIAN TRANSLATION 1**	
Sono in grado di seguire i miei progetti di lungo termine, anche quando il progresso è lento	
**ITALIAN TRANSLATION 2**	
Sono in grado di perseguire i miei piani a lungo termine anche se a volte procedo lentamente	
**RECONCILED ITALIAN TRANSLATION**	
Sono in grado di perseguire i miei piani a lungo termine anche quando procedono lentamente	
**BACKWARD TRANSLATION**	
I am able to pursue my long-term plans even when things move slowly	
**ADVANCED ITALIAN VERSION 1**	Reconciled OK. No comments from the ACT expert.
Sono in grado di perseguire i miei piani a lungo termine anche quando procedono lentamente	

ADVANCED ITALIAN VERSION 2 (post discussion with ACT expert)	
Sono in grado di perseguire i miei piani a lungo termine anche quando procedono lentamente	
**FINAL ITALIAN TRANSLATION**	
Sono in grado di perseguire i miei piani a lungo termine anche quando procedono lentamente	

ITEM # 18

ORIGINAL ENGLISH WORDING	COMMENTS/QUESTIONS
Even when something is important to me, I'll rarely do it if there is a chance it will upset me	
**ITALIAN TRANSLATION 1**	
Anche quando una cosa per me è importante, raramente la faccio se c'è il rischio che mi faccia star male	
**ITALIAN TRANSLATION 2**	
Anche se qualcosa è importante per me, difficilmente la faccio se c'è l'eventualità che io resti turbato	
**RECONCILED ITALIAN TRANSLATION**	
Anche quando una cosa è importante per me, difficilmente la faccio se c'è il rischio che mi faccia star male	
**BACKWARD TRANSLATION**	
Even when something matters to me, I tend not to do it if there is a risk that it might upset me	
**ADVANCED ITALIAN VERSION 1**	Reconciled OK. No comments from the ACT expert.
Anche quando una cosa è importante per me, difficilmente la faccio se c'è il rischio che mi faccia star male	
**ADVANCED ITALIAN VERSION 2 (post discussion with ACT expert)**	
Anche quando una cosa è importante per me, difficilmente la faccio se c'è il rischio che mi faccia star male	
**FINAL ITALIAN TRANSLATION**	
Anche quando una cosa è importante per me, difficilmente la faccio se c'è il rischio che mi faccia star male	

ITEM # 19

ORIGINAL ENGLISH WORDING	COMMENTS/QUESTIONS
It seems I am "running on automatic" without much awareness of what I'm doing	
**ITALIAN TRANSLATION 1**	
Mi sembra di fare le cose con il “pilota automatico”, senza rendermi bene conto di quello che faccio	
**ITALIAN TRANSLATION 2**	
Sembra che io proceda in modo automatico, senza essere molto consapevole di quello che sto facendo	
**RECONCILED ITALIAN TRANSLATION**	
Mi sembra di fare le cose con il “pilota automatico”, senza rendermi bene conto di quello che sto facendo	
**BACKWARD TRANSLATION**	
I seem to do things on “autopilot”, without actually being aware of what I am doing	
**ADVANCED ITALIAN VERSION 1**	Reconciled OK. No comments from the ACT expert.
Mi sembra di fare le cose con il “pilota automatico”, senza rendermi bene conto di quello che sto facendo	
**ADVANCED ITALIAN VERSION 2 (post discussion with ACT expert)**	
Mi sembra di fare le cose con il “pilota automatico”, senza rendermi bene conto di quello che sto facendo	
**FINAL ITALIAN TRANSLATION**	
Mi sembra di fare le cose con il “pilota automatico”, senza rendermi bene conto di quello che sto facendo	

ITEM # 20

ORIGINAL ENGLISH WORDING	COMMENTS/QUESTIONS
Thoughts are just thoughts – they don’t control what I do	
**ITALIAN TRANSLATION 1**	
I pensieri sono solo pensieri: non sono padroni di quello che faccio	
**ITALIAN TRANSLATION 2**	
I pensieri sono solo pensieri - non controllano quello che io faccio	
**RECONCILED ITALIAN TRANSLATION**	
I pensieri sono solo pensieri: non controllano quello che faccio	
**BACKWARD TRANSLATION**	
Thoughts are just thoughts: they do not control what I do	
**ADVANCED ITALIAN VERSION 1**	Reconciled OK. No comments from the ACT expert.

I pensieri sono solo pensieri: non controllano quello che faccio	
**ADVANCED ITALIAN VERSION 2 (post discussion with ACT expert)**	
I pensieri sono solo pensieri: non controllano quello che faccio	
**FINAL ITALIAN TRANSLATION**	
I pensieri sono solo pensieri: non controllano quello che faccio	

ITEM # 21

**ORIGINAL ENGLISH WORDING**	**COMMENTS/QUESTIONS**
My values are really reflected in my behaviour	
**ITALIAN TRANSLATION 1**	
Il mio comportamento rispecchia effettivamente i miei valori	
**ITALIAN TRANSLATION 2**	
I miei valori si rispecchiano concretamente nel mio comportamento	
**RECONCILED ITALIAN TRANSLATION**	
I miei valori si rispecchiano concretamente nel mio comportamento	
**BACKWARD TRANSLATION**	*I was worried about the beliefs/values switch here; however – 'valori' appears to translate back to values.*
My beliefs are accurately reflected in my behaviour	
**ADVANCED ITALIAN VERSION 1**	Reconciled OK.<br>I suggest to change "I miei valori di rispecchiano concretamente nel mio comportamento" with "Il mio comportamento rispecchia davvero I miei valori"
I miei valori si rispecchiano concretamente nel mio comportamento	
**ADVANCED ITALIAN VERSION 2 (post discussion with ACT expert)**	
Il mio comportamento rispecchia davvero i miei valori	
**FINAL ITALIAN TRANSLATION**	
Il mio comportamento rispecchia davvero i miei valori	

ITEM # 22

**ORIGINAL ENGLISH WORDING**	**COMMENTS/QUESTIONS**
I can take thoughts and feelings as they come, without attempting to control or avoid them	
**ITALIAN TRANSLATION 1**	
Posso prendere i miei pensieri e sentimenti così come sono, senza cercare di controllarli o di evitarli.	
**ITALIAN TRANSLATION 2**	

Sono in grado di accettare i pensieri e le emozioni per come vengono, senza provare a controllarli o a evitarli	
**RECONCILED ITALIAN TRANSLATION**	
Sono in grado di prendere pensieri ed emozioni così come vengono, senza cercare di controllarli o evitarli	
**BACKWARD TRANSLATION**	
I am able to take thoughts and emotions as they come, without trying to control or avoid them	
**ADVANCED ITALIAN VERSION 1**	Reconciled OK<br>I sugget to change “Sono in grado di prendere pensieri ed emozioni così come vengono, senza cercare di controllarli o evitarli” with “Posso accogliere i pensieri e le emozioni così come si presentano, senza cercare di controllarli o evitarli”
Sono in grado di prendere pensieri ed emozioni così come vengono, senza cercare di controllarli o evitarli	
**ADVANCED ITALIAN VERSION 2 (post discussion with ACT expert)**	
Sono in grado di accogliere pensieri e emozioni così come vengono, senza cercare di controllarli o evitarli	
**FINAL ITALIAN TRANSLATION**	
Sono in grado di accogliere pensieri e emozioni così come vengono, senza cercare di controllarli o evitarli	

ITEM # 23

**ORIGINAL ENGLISH WORDING**	**COMMENTS/QUESTIONS**
I can keep going with something when it’s important to me	
**ITALIAN TRANSLATION 1**	
Sono capace di tenere duro nelle cose che per me sono importanti.	
**ITALIAN TRANSLATION 2**	
Sono in grado di persistere con qualcosa quando questo è importante per me	
**RECONCILED ITALIAN TRANSLATION**	
Sono in grado di tenere duro quando qualcosa è importante per me	
**BACKWARD TRANSLATION**	
I am able to stick it out when something really matters to me	
**ADVANCED ITALIAN VERSION 1**	Reconciled OK<br>I suggest to change “tenere duro” with “andare avanti”,because “tenere duro” is a sort of resistence, a fight. The meaning here is passing through the experience and overcoming it by fully living it.
Sono in grado di tenere duro quando qualcosa è importante per me	

ADVANCED ITALIAN VERSION 2 (post discussion with ACT expert)	
Sono in grado di andare avanti quando qualcosa è importante per me	
**FINAL ITALIAN TRANSLATION**	
Sono in grado di andare avanti quando qualcosa è importante per me	
