# Supplementary material for: Cross-Country Adaptation of a Psychological Flexibility Measure: The Comprehensive Assessment of Acceptance and Commitment Therapy Processes
Source: Int J Environ Res Public Health. 2022 Mar 8;19(6):3150. doi: 10.3390/ijerph19063150 (PMC8953951; doi:10.3390/ijerph19063150)
Supplement: Supplementary file 1 [file ijerph-19-03150-s001.zip › Supplementary File 2 - German CompACT Transaltion Grid.pdf]

Article title: Cross-country adaptation of a psychological flexibility measure: The Comprehensive assessment of Acceptance and Commitment Therapy processes

Authors: Ambra Mara Giovannetti, Jana Pöttgen, Elisenda Anglada, Rebeca Menendez, Jürgen Hoyer, Andrea Giordano, Kenneth Ian Pakenham, Ingrid Galán, Alessandra Solari

Corresponding author: Ambra Mara Giovannetti, ambra.giovannetti@istituto-besta.it
Fondazione IRCCS Istituto Neurologico Carlo Besta, Milan, Italy. Via Celoria 11, 20133 Milano, Italia

Supplementary File 2 "German CompACT Translation grid"

COMMENTS/QUESTIONS MADE BY:
Please, use the appropriate colour based on this legenda.<br>[SMG] (Consultant German translator 1)<br>[IS] (Consultant German translator 2)<br>[YB] (Consultant backward translator)<br><br>[JP] (Researcher 1)<br>[CH] (Researcher2)<br>[NT] (Physician)<br>[KW] (Lay person)<br>[JH] (ACT expert)

TITLE

ORIGINAL ENGLISH WORDING	COMMENTS/QUESTIONS
CompACT (Comprehensive assessment of Acceptance and Commitment Therapy processes)	
**GERMAN TRANSLATION 1**	
CompACT (umfassendes Instrument zur Erfassung von Akzeptanz und Commitment basierten Therapieprozessen)	
**GERMAN TRANSLATION 2**	
CompACT (Umfassende Bewertung von Prozessen in der Akzeptanz- und Commitmenttherapie)	
**RECONCILIED GERMAN TRANSLATION**	
COMPACT (INSTRUMENT ZUR ERFASSUNG VON AKZEPTANZ UND COMMITMENT THERAPIEPROZESSEN)	
**BACKWARD**	
COMPACT (INSTRUMENT FOR THE ASSESSMENT OF ACCEPTANCE AND COMMITMENT THERAPY PROCESSES)	
**ADVANCED GERMAN VERSION 1**	Reconciled OK. No comments from the ACT expert.
CompACT (Comprehensive assessment of Acceptance and Commitment Therapy processes)	
**ADVANCED GERMAN VERSION 2 (post discussion with ACT expert)**	
CompACT (Comprehensive assessment of Acceptance and Commitment Therapy processes)	

FINAL GERMAN TRANSLATION	
CompACT (Comprehensive assessment of Acceptance and Commitment Therapy processes)	

INTRODUCTION

ORIGINAL ENGLISH WORDING	COMMENTS/QUESTIONS
Please rate the following 23 statements using the scale below:	
**GERMAN TRANSLATION 1**	
Bitte schätzen Sie die folgenden 23 Aussagen für sich gemäß folgender Skala ein!	
**GERMAN TRANSLATION 2**	
Bitte bewerten Sie die folgenden 23 Aussagen anhand der folgenden Skala:	
**RECONCILIED GERMAN TRANSLATION**	
Bitte bewerten Sie die folgenden 23 Aussagen anhand der folgenden Skala:	
**BACKWARD TRANSLATION**	
Please rate the following 23 statements using the following scale:	
**ADVANCED GERMAN VERSION 1**	Reconciled OK. No comments from the ACT expert.
Bitte bewerten Sie die folgenden 23 Aussagen anhand der folgenden Skala:	
**ADVANCED GERMAN VERSION 2 (post discussion with ACT expert)**	
Bitte bewerten Sie die folgenden 23 Aussagen anhand der folgenden Skala:	
**FINAL GERMAN TRANSLATION**	
Bitte bewerten Sie die folgenden 23 Aussagen anhand der folgenden Skala:	

POSSIBLE REPLY # 1

ORIGINAL ENGLISH WORDING	COMMENTS/QUESTIONS
Strongly disagree	
**GERMAN TRANSLATION 1**	Die wörtliche Übersetzung wäre eher “Lehne stark/mäßig/leicht ab.” “Stimme leicht/mäßig/sehr zu”, in Fragebögen wird aber standardmäßig eher die Stimme überhaupt nicht zu – nicht zu– eher nicht zu – eher zu – zu - voll und ganz zu.” verwendet, sodass ich dafür plädieren würde.<br><br>“The literal translation would be more like "reject Strong / moderate / light" Or like “Agree slightly / moderately / very much”. Usually, questionnaires tend to use “disagree at all - not agree - agree - completely agree." so that I would prefer this.”
Stimme gar nicht zu	
**GERMAN TRANSLATION 2**	
Stimme überhaupt nicht zu.	

**RECONCILIED GERMAN TRANSLATION**	
Stimme gar nicht zu	
**BACKWARD TRANSLATION**	
Totally disagree	
**ADVANCED GERMAN VERSION 1**	Reconciled OK. No comments from the ACT expert.
Starke Ablehnung	
**ADVANCED GERMAN VERSION 2 (post discussion with ACT expert)**	
Starke Ablehnung	
**FINAL GERMAN TRANSLATION**	
Starke Ablehnung	

POSSIBLE REPLY # 2

ORIGINAL ENGLISH WORDING	COMMENTS/QUESTIONS
Moderately disagree	
**GERMAN TRANSLATION 1**	
Stimme weitgehend nicht zu	
**GERMAN TRANSLATION 2**	
Stimme nicht zu.	
**RECONCILIED GERMAN TRANSLATION**	
Stimme weitgehend nicht zu	
**BACKWARD TRANSLATION**	
Largely disagree	
**ADVANCED GERMAN VERSION 1**	Reconciled OK. No comments from the ACT expert.
Mäßige Ablehnung	
**ADVANCED GERMAN VERSION 2 (post discussion with ACT expert)**	
Mäßige Ablehnung	
**FINAL GERMAN TRANSLATION**	
Mäßige Ablehnung	

POSSIBLE REPLY # 3

ORIGINAL ENGLISH WORDING	COMMENTS/QUESTIONS
Slightly disagree	
**GERMAN TRANSLATION 1**	

Stimme kaum zu	
**GERMAN TRANSLATION 2**	
Stimme eher nicht zu.	
**RECONCILIED GERMAN TRANSLATION**	
Stimme eher nicht zu.	
**BACKWARD TRANSLATION**	
Rather disagree	
**ADVANCED GERMAN VERSION 1**	Reconciled OK. No comments from the ACT expert.
Geringe Ablehnung	
**ADVANCED GERMAN VERSION 2 (post discussion with ACT expert)**	
Geringe Ablehnung	
**FINAL GERMAN TRANSLATION**	
Geringe Ablehnung	

POSSIBLE REPLY # 4

ORIGINAL ENGLISH WORDING	COMMENTS/QUESTIONS
Neither agree nor disagree	
**GERMAN TRANSLATION 1**	
Neutral	
**GERMAN TRANSLATION 2**	Die wörtliche Übersetzung klingt ziemlich holprig (und verwirrt evtl. Auch durch das "lehne ab", was sonst nicht vorkommt). Alternativ: "Weder noch." Oder "Neutral".<br>The literal translation sounds a bit bumpy (and may also confuse by the "reject", which usually does not occur). Alternatively: "Neither nor" Or "neutral".
Stimme weder zu noch lehne ich ab.	
**RECONCILIED GERMAN TRANSLATION**	
Weder noch	
**BACKWARD TRANSLATION**	
Neither nor	
**ADVANCED GERMAN VERSION 1**	Reconciled OK. No comments from the ACT expert.
Weder Zustimmung noch Ablehnung	
**ADVANCED GERMAN VERSION 2 (post discussion with ACT expert)**	
Weder Zustimmung noch Ablehnung	
**FINAL GERMAN TRANSLATION**	

Weder Zustimmung noch Ablehnung	

POSSIBLE REPLY # 5

ORIGINAL ENGLISH WORDING	COMMENTS/QUESTIONS
Slightly agree	
**GERMAN TRANSLATION 1**	
Stimme ein bisschen zu	
**GERMAN TRANSLATION 2**	
Stimme eher zu.	
**RECONCILIED GERMAN TRANSLATION**	
Stimme eher zu.	
**BACKWARD TRANSLATION**	
Rather agree	
**ADVANCED GERMAN VERSION 1**	Reconciled OK. No comments from the ACT expert.
Geringe Zustimmung	
**ADVANCED GERMAN VERSION 2 (post discussion with ACT expert)**	
Geringe Zustimmung	
**FINAL GERMAN TRANSLATION**	
Geringe Zustimmung	

POSSIBLE REPLY # 6

ORIGINAL ENGLISH WORDING	COMMENTS/QUESTIONS
Moderately agree	
**GERMAN TRANSLATION 1**	
Stimme weitgehend zu	
**GERMAN TRANSLATION 2**	
Stimme zu.	
**RECONCILIED GERMAN TRANSLATION**	
Stimme weitgehend zu	
**BACKWARD TRANSLATION**	
Largely agree	
**ADVANCED GERMAN VERSION 1**	Reconciled OK. No comments from the ACT expert.
Mäßige Zustimmung	
**ADVANCED GERMAN VERSION 2 (post discussion with ACT expert)**	

Mäßige Zustimmung	
**FINAL GERMAN TRANSLATION**	
Mäßige Zustimmung	

POSSIBLE REPLY # 7

ORIGINAL ENGLISH WORDING	COMMENTS/QUESTIONS
Strongly agree	
**GERMAN TRANSLATION 1**	
Stimme vollkommen zu	
**GERMAN TRANSLATION 2**	
Stimme voll und ganz zu.	
**RECONCILIED GERMAN TRANSLATION**	
Stimme voll und ganz zu.	
**BACKWARD TRANSLATION**	
Totally agree	
**ADVANCED GERMAN VERSION 1**	Reconciled OK. No comments from the ACT expert.
Starke Zustimmung	
**ADVANCED GERMAN VERSION 2 (post discussion with ACT expert)**	
Starke Zustimmung	
**FINAL GERMAN TRANSLATION**	
Starke Zustimmung	

ITEM # 1

ORIGINAL ENGLISH WORDING	COMMENTS/QUESTIONS
I can identify the things that really matter to me in life and pursue them	
**GERMAN TRANSLATION 1**	
Ich kann Dinge benennen, die mir im Leben wirklich wichtig sind, *und diese auch gezielt anstreben.*	
**GERMAN TRANSLATION 2**	Instead of “identifizieren” I suggest to use “erkennen” that is simpler German.
Ich kann die Dinge, die mir im Leben wirklich wichtig sind identifizieren und sie verfolgen.	
**RECONCILIED GERMAN TRANSLATION**	
Ich kann Dinge benennen, die mir im Leben wirklich wichtig sind, *und diese auch gezielt anstreben.*	
**BACKWARD TRANSLATION**	

I can name things that are really important to me in life and I can also strive for these goals.	
**ADVANCED GERMAN VERSION 1**	JH: I thing “bennen” is not the correct translation of "identify". Since it is about psychological processes, "recognizing" ("erkennen" in German) might be better.<br><br>JP: We discussed it in our group and decided to keep using “bennen” instead of "erkennen" because we felt it is closer to our common language and more understandable for people with no psychological background
Ich kann Dinge benennen, die mir im Leben wirklich wichtig sind, und diese auch verfolgen.	
**ADVANCED GERMAN VERSION 2 (post discussion with ACT expert)**	
Ich kann Dinge benennen, die mir im Leben wirklich wichtig sind, und diese auch verfolgen.	
**FINAL GERMAN TRANSLATION**	
Ich kann Dinge benennen, die mir im Leben wirklich wichtig sind, und diese auch verfolgen.	

ITEM # 2

ORIGINAL ENGLISH WORDING	COMMENTS/QUESTIONS
One of my big goals is to be free from painful emotions	
**GERMAN TRANSLATION 1**	
Eins meiner großen Ziele ist es, schmerzvolle Gefühle möglichst zu vermeiden.	
**GERMAN TRANSLATION 2**	
Eines meiner großen Ziele ist es, frei von schmerzlichen Gefühlen zu sein.	
**RECONCILIED GERMAN TRANSLATION**	
Eines meiner großen Ziele ist es, keine schmerzhaften Gefühle zu haben.	
**BACKWARD TRANSLATION**	
One of my major aims is to have no painful feelings	
**ADVANCED GERMAN VERSION 1**	Reconciled OK. No comments from the ACT expert.
Eines meiner großen Ziele ist es, keine schmerzhaften Gefühle zu haben.	
**ADVANCED GERMAN VERSION 2 (post discussion with ACT expert)**	
Eines meiner großen Ziele ist es, keine schmerzhaften Gefühle zu haben.	
**FINAL GERMAN TRANSLATION**	
Eines meiner großen Ziele ist es, keine schmerzhaften Gefühle zu haben.	

ITEM # 3

ORIGINAL ENGLISH WORDING	COMMENTS/QUESTIONS
I rush through meaningful activities without being really attentive to them	

GERMAN TRANSLATION 1	
Oft haste ich durch *bedeutsame Aktivitäten,* ohne sie wirklich bewusst zu erleben.	
**GERMAN TRANSLATION 2**	
Ich eile durch bedeutungsvolle Unternehmungen, ohne wirklich auf sie zu achten.	
**RECONCILIED GERMAN TRANSLATION**	
Oft haste ich durch *bedeutsame Aktivitäten,* ohne sie wirklich bewusst zu erleben.	
**BACKWARD TRANSLATION**	
I often rush through meaningful activities without really experiencing them consciously.	
**ADVANCED GERMAN VERSION 1**	Reconciled OK. No comments from the ACT expert.
Ich haste durch bedeutsame Aktivitäten, ohne sie wirklich bewusst zu erleben.	
**ADVANCED GERMAN VERSION 2 (post discussion with ACT expert)**	
Ich haste durch bedeutsame Aktivitäten, ohne sie wirklich bewusst zu erleben.	
**FINAL GERMAN TRANSLATION**	
Ich haste durch bedeutsame Aktivitäten, ohne sie wirklich bewusst zu erleben.	

ITEM # 4

ORIGINAL ENGLISH WORDING	COMMENTS/QUESTIONS
I try to stay busy to keep thoughts or feelings from coming	
**GERMAN TRANSLATION 1**	
Ich versuche stets beschäftigt zu bleiben, um keine Gedanken oder Gefühle aufkommen zu lassen.	
**GERMAN TRANSLATION 2**	The literal translation would be "... to keep feelings from coming up." - that sounds strange in German.<br>Die wörtliche Übersetung ware "…Gefühle davon abzuhalten, zu kommen." – das klingt auf deutsch aber komisch.
Ich versuche, ständig beschäftigt zu bleiben, um Gedanken oder Gefühle fern zu halten.	
**RECONCILIED GERMAN TRANSLATION**	
Ich versuche stets beschäftigt zu sein, um keine Gedanken oder Gefühle aufkommen zu lassen.	
**BACKWARD TRANSLATION**	
I always try to keep busy so as not to let any thoughts or feelings arise.	
**ADVANCED GERMAN VERSION 1**	Reconciled OK. No comments from the ACT expert.
Ich versuche stets beschäftigt zu sein, um keine Gedanken oder Gefühle aufkommen zu lassen.	
**ADVANCED GERMAN VERSION 2 (post discussion with ACT expert)**	
Ich versuche stets beschäftigt zu sein, um keine Gedanken oder Gefühle aufkommen zu	

lassen.	
**FINAL GERMAN TRANSLATION**	
Ich versuche stets beschäftigt zu sein, um keine Gedanken oder Gefühle aufkommen zu lassen.	

ITEM # 5

**ORIGINAL ENGLISH WORDING**	**COMMENTS/QUESTIONS**
I act in ways that are consistent with how I wish to live my life	
**GERMAN TRANSLATION 1**	
Ich verhalte mich so, wie es meiner Art zu leben entspricht.	
**GERMAN TRANSLATION 2**	
Ich handle in einer Art und Weise, die damit überein stimmt, wie ich mein Leben leben möchte.	
**RECONCILIED GERMAN TRANSLATION**	
Ich handle so, wie es meiner Art zu Leben entspricht.	
**BACKWARD TRANSLATION**	
I act in accordance with my way of life.	
**ADVANCED GERMAN VERSION 1**	Reconciled OK. No comments from the ACT expert.
Ich lebe so, wie ich mein Leben leben möchte.	
**ADVANCED GERMAN VERSION 2 (post discussion with ACT expert)**	
Ich lebe so, wie ich mein Leben leben möchte.	
**FINAL GERMAN TRANSLATION**	
Ich lebe so, wie ich mein Leben leben möchte.	

ITEM # 6

**ORIGINAL ENGLISH WORDING**	**COMMENTS/QUESTIONS**
I get so caught up in my thoughts that I am unable to do the things that I most want to do	
**GERMAN TRANSLATION 1**	
Ich verfange mich so sehr in meinen Gedanken, dass es mich davon abhält, die Dinge zu tun, die mir am wichtigsten sind.	
**GERMAN TRANSLATION 2**	Alternativ: “Ich stecke so in meinen Gedanken fest, …” Und für das Ende alternativ “… Dinge zu tun, die mir am wichtigsten sind.” Alternatively: "I'm stucked in my thoughts ..." And alternatively, for the end, “... doing things that are most
Ich bin so in meinen Gedanken gefangen, dass ich nicht in der Lage bin, die Dinge zu tun, die ich am meisten tun möchte.	

	important to me.”
**RECONCILIED GERMAN TRANSLATION**	
Ich bin so in meinen Gedanken gefangen, dass es mich davon abhält, die Dinge zu tun, die mir am wichtigsten sind.	
**BACKWARD TRANSLATION**	
I’m so caught up in my thoughts that it keeps me from doing the things that are most important to me.	
**ADVANCED GERMAN VERSION 1**	JH: "die ich am liebsten tun möchte" could be interpreted as "am wichtigsten sind".<br><br>JP: here we decided to stay as close as possible to the original english version. “Want to do” does not really mean “important“. We interpreted it like an internal desire.
Ich bin so in meinen Gedanken gefangen, dass es mich davon abhält, die Dinge zu tun, die ich am liebsten tun möchte.	
**ADVANCED GERMAN VERSION 2 (post discussion with ACT expert)**	
Ich bin so in meinen Gedanken gefangen, dass es mich davon abhält, die Dinge zu tun, die ich am liebsten tun möchte.	
**FINAL GERMAN TRANSLATION**	
Ich bin so in meinen Gedanken gefangen, dass es mich davon abhält, die Dinge zu tun, die ich am liebsten tun möchte.	

ITEM # 7

ORIGINAL ENGLISH WORDING	COMMENTS/QUESTIONS
I make choices based on what is important to me, even if it is stressful	
**GERMAN TRANSLATION 1**	
Ich treffe Entscheidungen entsprechend dessen was mir wichtig ist, auch wenn es *schwierig/belastend* ist.	
**GERMAN TRANSLATION 2**	
Ich treffe Entscheidungen auf der Grundlage dessen, was mir wichtig ist, auch wenn es stressig ist.	
**RECONCILIED GERMAN TRANSLATION**	
Ich treffe Entscheidungen danach, was mir wichtig ist, auch wenn es mich *belastet*.	
**BACKWARD TRANSLATION**	
I make decisions based on what is important to me even if it stresses me.	
**ADVANCED GERMAN VERSION 1**	Reconciled OK. No comments from the ACT expert.
Ich treffe Entscheidungen danach, was mir wichtig ist, auch wenn es mich belastet.	

ADVANCED GERMAN VERSION 2 (post discussion with ACT expert)	
Ich treffe Entscheidungen danach, was mir wichtig ist, auch wenn es mich belastet.	
**FINAL GERMAN TRANSLATION**	
Ich treffe Entscheidungen danach, was mir wichtig ist, auch wenn es mich belastet.	

ITEM # 8

**ORIGINAL ENGLISH WORDING**	**COMMENTS/QUESTIONS**
I tell myself that I shouldn’t have certain thoughts	
**GERMAN TRANSLATION 1**	
Ich verbiete mir bestimmte Gedanken.	
**GERMAN TRANSLATION 2**	
Ich sage mir, dass ich bestimmte Gedanken nicht haben sollte.	
**RECONCILIED GERMAN TRANSLATION**	
Ich sage mir, dass ich bestimmte Gedanken nicht haben sollte.	
**BACKWARD TRANSLATION**	
I tell myself I shouldn't have certain thoughts.	
**ADVANCED GERMAN VERSION 1**	Reconciled OK. No comments from the ACT expert.
Ich sage mir, dass ich bestimmte Gedanken nicht haben sollte.	
**ADVANCED GERMAN VERSION 2 (post discussion with ACT expert)**	
Ich sage mir, dass ich bestimmte Gedanken nicht haben sollte.	
**FINAL GERMAN TRANSLATION**	
Ich sage mir, dass ich bestimmte Gedanken nicht haben sollte.	

ITEM # 9

**ORIGINAL ENGLISH WORDING**	**COMMENTS/QUESTIONS**
I find it difficult to stay focused on what’s happening in the present	
**GERMAN TRANSLATION 1**	
Ich finde es schwierig mich darauf zu konzentrieren, was in einem Moment gerade geschieht.	
**GERMAN TRANSLATION 2**	Alternativ statt “in der Gegenwart” – “gerade”<br>Alternatively instead of “in the present” - “just”
Ich finde es schwierig, mich auf das zu konzentrieren, was in der Gegenwart passiert.	
**RECONCILIED GERMAN TRANSLATION**	
Ich finde es schwierig, mich darauf zu konzentrieren, was in einem Moment gerade	

geschieht.	
**BACKWARD TRANSLATION**	
I find it difficult to concentrate on what's going on at a particular moment.	
**ADVANCED GERMAN VERSION 1**	Reconciled OK. No comments from the ACT expert.
Es fällt mir schwer, mich auf das zu konzentrieren, was gerade geschieht.	
**ADVANCED GERMAN VERSION 2 (post discussion with ACT expert)**	
Es fällt mir schwer, mich auf das zu konzentrieren, was gerade geschieht.	
**FINAL GERMAN TRANSLATION**	
Es fällt mir schwer, mich auf das zu konzentrieren, was gerade geschieht.	

ITEM # 10

ORIGINAL ENGLISH WORDING	COMMENTS/QUESTIONS
I behave in line with my personal values	
**GERMAN TRANSLATION 1**	
Ich verhalte mich so, wie es meinen Werten entspricht.	
**GERMAN TRANSLATION 2**	
Ich handele im Einklang mit meinen persönlichen Werten.	
**RECONCILIED GERMAN TRANSLATION**	
Ich handle im Einklang mit meinen persönlichen Werten.	
**BACKWARD TRANSLATION**	
I act in accordance with my personal values.	
**ADVANCED GERMAN VERSION 1**	Reconciled OK. No comments from the ACT expert.
Ich handle im Einklang mit meinen persönlichen Werten.	
**ADVANCED GERMAN VERSION 2 (post discussion with ACT expert)**	
Ich handle im Einklang mit meinen persönlichen Werten.	
**FINAL GERMAN TRANSLATION**	
Ich handle im Einklang mit meinen persönlichen Werten.	

ITEM # 11

ORIGINAL ENGLISH WORDING	COMMENTS/QUESTIONS
I go out of my way to avoid situations that might bring difficult thoughts, feelings, or sensations	
**GERMAN TRANSLATION 1**	
Ich unternehme besondere Anstrengungen um Situationen zu vermeiden, in denen schwierige Gedanken, Gefühle oder Empfindungen aufkommen könnten.	

**GERMAN TRANSLATION 2**	
Ich versuche, Situationen zu vermeiden, die schwierige Gedanken, Gefühle oder Empfindungen hervorrufen könnten.	
**RECONCILIED GERMAN TRANSLATION**	
Ich versuche alles, um Situationen zu vermeiden, die schwierige Gedanken, Gefühle oder Empfindungen hervorrufen könnten.	
**BACKWARD TRANSLATION**	
I do all I can to avoid situations that could give rise to difficult thoughts, feelings or emotions.	
**ADVANCED GERMAN VERSION 1**	Reconciled OK. No comments from the ACT expert.
Ich versuche alles, um Situationen zu vermeiden, die schwierige Gedanken, Gefühle oder Empfindungen hervorrufen könnten.	
**ADVANCED GERMAN VERSION 2 (post discussion with ACT expert)**	
Ich versuche alles, um Situationen zu vermeiden, die schwierige Gedanken, Gefühle oder Empfindungen hervorrufen könnten.	
**FINAL GERMAN TRANSLATION**	
Ich versuche alles, um Situationen zu vermeiden, die schwierige Gedanken, Gefühle oder Empfindungen hervorrufen könnten.	

TEM # 12

ORIGINAL ENGLISH WORDING	COMMENTS/QUESTIONS
Even when doing the things that matter to me, I find myself doing them without paying attention	
**GERMAN TRANSLATION 1**	
Selbst wenn ich Dinge tue, die *mir wichtig / für mich bedeutsam* sind, *erlebe ich diese oft nicht bewusst.*	
**GERMAN TRANSLATION 2**	Alternativ "… nicht ganz bei der Sache bin" oder "… nicht richtig aufpasse"<br><br>Alternatively, "… I'm not quite there" or "… not really paying attention"
Selbst wenn ich Dinge tue, die mir wichtig sind, merke ich, dass ich nicht wirklich Acht gebe.	
**RECONCILIED GERMAN TRANSLATION**	
Selbst wenn ich Dinge tue, die mir wichtig sind, wird mir manchmal bewusst, dass ich nicht bei der Sache bin.	
**BACKWARD TRANSLATION**	

Even when I'm doing things that are important to me I sometimes realise that my mind is not on them.	
**ADVANCED GERMAN VERSION 1**	JH: "wird mir bewusst", is at least as good as: "bemerke ich"
Selbst wenn ich Dinge tue, die mir wichtig sind, wird mir bewusst, dass ich nicht bei der Sache bin.	
**ADVANCED GERMAN VERSION 2 (post discussion with ACT expert)**	
Selbst wenn ich Dinge tue, die mir wichtig sind, merke ich, dass ich nicht bei der Sache bin.	
**FINAL GERMAN TRANSLATION**	
Selbst wenn ich Dinge tue, die mir wichtig sind, merke ich, dass ich nicht bei der Sache bin.	

ITEM # 13

ORIGINAL ENGLISH WORDING	COMMENTS/QUESTIONS
I am willing to fully experience whatever thoughts, feelings and sensations come up for me, without trying to change or defend against them	
**GERMAN TRANSLATION 1**	
Ich bin bereit mich auf alle aufkommenden Gedanken, Gefühle und Empfindungen voll einzulassen, ohne sie beeinflussen oder verhindern zu *versuchen/wollen*.	
**GERMAN TRANSLATION 2**	
Ich bin bereit, alle Gedanken, Gefühle und Empfindungen, die in mir aufkommen, vollständig zuzulassen, ohne zu versuchen, sie zu ändern oder abzuwehren.	
**RECONCILIED GERMAN TRANSLATION**	
Ich bin bereit, mich auf alle aufkommenden Gedanken, Gefühle und Empfindungen voll einzulassen, ohne zu versuchen, sie zu ändern oder abzuwehren.	
**BACKWARD TRANSLATION**	
I am ready to engage fully with all emerging thoughts, feelings and emotions without trying to change or repel them.	
**ADVANCED GERMAN VERSION 1**	Reconciled OK. No comments from the ACT expert.
Ich bin bereit, alle aufkommenden Gedanken, Gefühle und Empfindungen voll zuzulassen, ohne zu versuchen, sie zu ändern oder abzuwehren.	
**ADVANCED GERMAN VERSION 2 (post discussion with ACT expert)**	
Ich bin bereit, alle aufkommenden Gedanken, Gefühle und Empfindungen voll zuzulassen, ohne zu versuchen, sie zu ändern oder abzuwehren.	
**FINAL GERMAN TRANSLATION**	
Ich bin bereit, alle aufkommenden Gedanken, Gefühle und Empfindungen voll zuzulassen, ohne zu versuchen, sie zu ändern oder abzuwehren.	

ITEM # 14

ORIGINAL ENGLISH WORDING	COMMENTS/QUESTIONS
I undertake things that are meaningful to me, even when I find it hard to do so	
**GERMAN TRANSLATION 1**	
Ich nehme Dinge in Angriff, die für mich bedeutsam sind, auch wenn das für mich schwierig ist.	
**GERMAN TRANSLATION 2**	
Ich unternehme Dinge, die für mich von Bedeutung sind, auch wenn es mir schwer fällt, dies zu tun.	
**RECONCILIED GERMAN TRANSLATION**	
Ich nehme Dinge in Angriff, die für mich bedeutsam sind, auch wenn es mir schwer fällt.	
**BACKWARD TRANSLATION**	
I tackle things that matter to me even if it's hard for me.	
**ADVANCED GERMAN VERSION 1**	Reconciled OK. No comments from the ACT expert.
Ich nehme Dinge in Angriff, die für mich bedeutsam sind, auch wenn es mir schwer fällt.	
**ADVANCED GERMAN VERSION 2 (post discussion with ACT expert)**	
Ich nehme Dinge in Angriff, die für mich bedeutsam sind, auch wenn es mir schwer fällt.	
**FINAL GERMAN TRANSLATION**	
Ich nehme Dinge in Angriff, die für mich bedeutsam sind, auch wenn es mir schwer fällt.	

ITEM # 15

ORIGINAL ENGLISH WORDING	COMMENTS/QUESTIONS
I work hard to keep out upsetting feelings	
**GERMAN TRANSLATION 1**	
Ich bemühe mich sehr, unangenehme Gefühle *nicht zuzulassen*.	
**GERMAN TRANSLATION 2**	
Ich verwende viel Kraft darauf, beunruhigende Gefühle fernzuhalten.	
**RECONCILIED GERMAN TRANSLATION**	
Ich bemühe mich sehr darum belastende Gefühle fernzuhalten.	
**BACKWARD TRANSLATION**	
I try very hard to keep stressful feelings away.	
**ADVANCED GERMAN VERSION 1**	Reconciled OK. No comments from the ACT expert.
Ich bemühe mich sehr, beunruhigende Gefühle fernzuhalten.	
**ADVANCED GERMAN VERSION 2 (post discussion with ACT expert)**	

Ich bemühe mich sehr, beunruhigende Gefühle fernzuhalten.	
**FINAL GERMAN TRANSLATION**	
Ich bemühe mich sehr, beunruhigende Gefühle fernzuhalten.	

ITEM # 16

**ORIGINAL ENGLISH WORDING**	**COMMENTS/QUESTIONS**
I do jobs or tasks automatically, without being aware of what I'm doing	
**GERMAN TRANSLATION 1**	
Ich erledige Aufgaben und Tätigkeiten automatisch, ohne mir recht bewusst zu sein was ich gerade tue.	
**GERMAN TRANSLATION 2**	
Ich erledige Aufgaben automatisch, ohne dass mir bewusst ist, was ich tue.	
**RECONCILIED GERMAN TRANSLATION**	
Ich erledige Aufgaben und Tätigkeiten automatisch, ohne mir recht bewusst zu sein, was ich gerade tue.	
**BACKWARD TRANSLATION**	
I deal with tasks and activities automatically without really being conscious of what I am doing at the time.	
**ADVANCED GERMAN VERSION 1**	Reconciled OK. No comments from the ACT expert.
Ich erledige Aufgaben und Tätigkeiten automatisch, ohne mir recht bewusst zu sein, was ich gerade tue.	
**ADVANCED GERMAN VERSION 2 (post discussion with ACT expert)**	
Ich erledige Aufgaben und Tätigkeiten automatisch, ohne mir recht bewusst zu sein, was ich gerade tue.	
**FINAL GERMAN TRANSLATION**	
Ich erledige Aufgaben und Tätigkeiten automatisch, ohne mir recht bewusst zu sein, was ich gerade tue.	

ITEM # 17

**ORIGINAL ENGLISH WORDING**	**COMMENTS/QUESTIONS**
I am able to follow my long term plans including times when progress is slow	
**GERMAN TRANSLATION 1**	
Ich kann an meinen langfristigen Zielen festhalten, auch wenn ich manchmal nur kleine Fortschritte mache.	
**GERMAN TRANSLATION 2**	

Ich bin in der Lage, meine langfristige Pläne zu verfolgen, auch dann, wenn es nur langsam voran geht.	
**RECONCILIED GERMAN TRANSLATION**	
Ich bin in der Lage, meine langfristigen Pläne weiter zu verfolgen, auch wenn es nur langsam voran geht.	
**BACKWARD TRANSLATION**	
I am able to pursue my long-term plans further even if progress is only slow.	
**ADVANCED GERMAN VERSION 1**	Reconciled OK. No comments from the ACT expert.
Ich bin in der Lage, meine langfristigen Pläne zu verfolgen, auch wenn es langsam voran geht.	
**ADVANCED GERMAN VERSION 2 (post discussion with ACT expert)**	
Ich bin in der Lage, meine langfristigen Pläne zu verfolgen, auch wenn es langsam voran geht.	
**FINAL GERMAN TRANSLATION**	
Ich bin in der Lage, meine langfristigen Pläne zu verfolgen, auch wenn es langsam voran geht.	

ITEM # 18

ORIGINAL ENGLISH WORDING	COMMENTS/QUESTIONS
Even when something is important to me, I'll rarely do it if there is a chance it will upset me	
**GERMAN TRANSLATION 1**	
Selbst wenn mir etwas sehr wichtig ist tue ich es eher nicht, wenn die Möglichkeit besteht dass es mich *beunruhigen/verunsichern* könnte.	
**GERMAN TRANSLATION 2**	"Upset" könnte auch als "verärgert sein" übersetzt warden. "Upset in the meaning of excited" could also be translated as "upset"
Selbst wenn mir etwas wichtig ist, tue ich es eher nicht, wenn die Chance besteht, dass es mich aufregen könnte.	
**RECONCILIED GERMAN TRANSLATION**	
Selbst wenn mir etwas wichtig ist, tue ich es eher nicht, wenn die Möglichkeit besteht, dass es mich *beunruhigen* könnte.	
**BACKWARD TRANSLATION**	
Even if something is important to me I tend not to do it if there is a possibility that it might unsettle me.	
**ADVANCED GERMAN VERSION 1**	JH: the translation is correct, but I would suggest to use "sofern" instead of "wenn"
Selbst wenn mir etwas wichtig ist, tue ich es eher nicht, wenn die Möglichkeit besteht, dass	

es mich beunruhigen könnte.	JP: we decided to use “wenn/if” because “sofern/unless” is very academic language
**ADVANCED GERMAN VERSION 2 (post discussion with ACT expert)**	
Selbst wenn mir etwas wichtig ist, tue ich es eher nicht, wenn die Möglichkeit besteht, dass es mich beunruhigen könnte	
**FINAL GERMAN TRANSLATION**	
Selbst wenn mir etwas wichtig ist, tue ich es eher nicht, wenn die Möglichkeit besteht, dass es mich beunruhigen könnte	

ITEM # 19

**ORIGINAL ENGLISH WORDING**	**COMMENTS/QUESTIONS**
It seems I am "running on automatic" without much awareness of what I'm doing	
**GERMAN TRANSLATION 1**	
Es kommt mir *(oft)* so vor als ob ich „automatische Programme“ abspule, ohne mir bewusst zu sein was ich gerade tue.	
**GERMAN TRANSLATION 2**	Etwas umgangssprachlicher würde ich es übersetzen als “Es kommt mir so vor, als ware ich auf “Autopilot” geschaltet, …” I would translate it a bit more colloquially as “It seems to me that I am on “autopilot”...”
Es kommt mir so vor, als wäre ich "auf Automatik" geschaltet, ohne dass ich bewusst wahrnehme, was ich tue.	
**RECONCILIED GERMAN TRANSLATION**	
Es kommt mir so vor, als wäre ich "auf Automatik" geschaltet, ohne dass ich bewusst wahrnehme, was ich tue.	
**BACKWARD TRANSLATION**	
It seems to me as if I’ve been “switched to automatic" without consciously experiencing what I’m doing.	
**ADVANCED GERMAN VERSION 1**	Reconciled OK. No comments from the ACT expert.
Es kommt mir so vor, als wäre ich "auf Automatik" geschaltet, ohne dass ich bewusst wahrnehme, was ich tue.	
**ADVANCED GERMAN VERSION 2 (post discussion with ACT expert)**	
Es kommt mir so vor, als wäre ich "auf Automatik" geschaltet, ohne dass ich bewusst wahrnehme, was ich tue.	
**FINAL GERMAN TRANSLATION**	
Es kommt mir so vor, als wäre ich "auf Automatik" geschaltet, ohne dass ich bewusst wahrnehme, was ich tue.	

ITEM # 20

ORIGINAL ENGLISH WORDING	COMMENTS/QUESTIONS
Thoughts are just thoughts – they don’t control what I do	
**GERMAN TRANSLATION 1**	
Gedanken sind nur Gedanken, sie bestimmen nicht was ich tue.	
**GERMAN TRANSLATION 2**	
Gedanken sind nur Gedanken - sie bestimmen nicht, was ich tue.	
**RECONCILIED GERMAN TRANSLATION**	
Gedanken sind nur Gedanken - sie bestimmen nicht was ich tue	
**BACKWARD TRANSLATION**	
Thoughts are just thoughts - they don't dictate what I do.	
**ADVANCED GERMAN VERSION 1**	Reconciled OK. No comments from the ACT expert.
Gedanken sind nur Gedanken - sie bestimmen nicht, was ich tue.	
**ADVANCED GERMAN VERSION 2 (post discussion with ACT expert)**	
Gedanken sind nur Gedanken - sie bestimmen nicht, was ich tue.	
**FINAL GERMAN TRANSLATION**	
Gedanken sind nur Gedanken - sie bestimmen nicht, was ich tue.	

ITEM # 21

ORIGINAL ENGLISH WORDING	COMMENTS/QUESTIONS
My values are really reflected in my behaviour	
**GERMAN TRANSLATION 1**	
Alle meine Werte spiegeln sich auch wirklich in meinem Verhalten wider.	
**GERMAN TRANSLATION 2**	
Meine Werte spiegeln sich wirklich in meinem Verhalten wider.	
**RECONCILIED GERMAN TRANSLATION**	
Alle meine Werte spiegeln sich auch wirklich in meinem Verhalten wider.	
**BACKWARD TRANSLATION**	
All my values are also genuinely reflected in my behaviour.	
**ADVANCED GERMAN VERSION 1**	Reconciled OK. No comments from the ACT expert.
Meine Werte spiegeln sich wirklich in meinem Verhalten wider.	
**ADVANCED GERMAN VERSION 2 (post discussion with ACT expert)**	
Meine Werte spiegeln sich wirklich in meinem Verhalten wider.	

FINAL GERMAN TRANSLATION	
Meine Werte spiegeln sich wirklich in meinem Verhalten wider.	

ITEM # 22

ORIGINAL ENGLISH WORDING	COMMENTS/QUESTIONS
I can take thoughts and feelings as they come, without attempting to control or avoid them	
**GERMAN TRANSLATION 1**	
Ich kann Gedanken und Gefühle so nehmen wie sie kommen, ohne zu versuchen sie zu kontrollieren oder zu vermeiden.	
**GERMAN TRANSLATION 2**	Alternativ "… sie zu beeinflussen oder zu vermeiden." Alternatively, "... to influence or avoid them."
Ich kann Gedanken und Gefühle so nehmen, wie sie kommen, ohne zu versuchen, sie zu kontrollieren oder zu vermeiden.	
**RECONCILIED GERMAN TRANSLATION**	
Ich kann Gedanken und Gefühle so nehmen, wie sie kommen, ohne zu versuchen, sie zu kontrollieren oder zu vermeiden.	
**BACKWARD TRANSLATION**	
I can take thoughts and feelings as they come without trying to control or avoid them.	
**ADVANCED GERMAN VERSION 1**	Reconciled OK. No comments from the ACT expert.
Ich kann Gedanken und Gefühle so nehmen, wie sie kommen, ohne zu versuchen, sie zu kontrollieren oder zu vermeiden.	
**ADVANCED GERMAN VERSION 2 (post discussion with ACT expert)**	
Ich kann Gedanken und Gefühle so nehmen, wie sie kommen, ohne zu versuchen, sie zu kontrollieren oder zu vermeiden.	
**FINAL GERMAN TRANSLATION**	
Ich kann Gedanken und Gefühle so nehmen, wie sie kommen, ohne zu versuchen, sie zu kontrollieren oder zu vermeiden.	

ITEM # 23

ORIGINAL ENGLISH WORDING	COMMENTS/QUESTIONS
I can keep going with something when it's important to me	
**GERMAN TRANSLATION 1**	
Ich kann an einer Sache weitermachen, wenn sie mir wichtig ist.	
**GERMAN TRANSLATION 2**	
Ich kann etwas durchziehen, wenn es mir wichtig ist.	
**RECONCILIED GERMAN TRANSLATION**	

Ich kann etwas durchziehen, wenn es mir wichtig ist.	
**BACKWARD TRANSLATION**	
I can follow something through if it's important to me.	
**ADVANCED GERMAN VERSION 1**	Reconciled OK. No comments from the ACT expert.
Ich kann an etwas dranbleiben, wenn es mir wichtig ist.	
**ADVANCED GERMAN VERSION 2 (post discussion with ACT expert)**	
Ich kann an etwas dranbleiben, wenn es mir wichtig ist.	
**FINAL GERMAN TRANSLATION**	
Ich kann an etwas dranbleiben, wenn es mir wichtig ist.	
