# Supplementary material for: Cross-Country Adaptation of a Psychological Flexibility Measure: The Comprehensive Assessment of Acceptance and Commitment Therapy Processes
Source: Int J Environ Res Public Health. 2022 Mar 8;19(6):3150. doi: 10.3390/ijerph19063150 (PMC8953951; doi:10.3390/ijerph19063150)
Supplement: Supplementary file 1 [file ijerph-19-03150-s001.zip › Supplementary File 3 - Spanish CompACT Translation Grid.doc.pdf]

Article title: Cross-country adaptation of a psychological flexibility measure: The Comprehensive assessment of Acceptance and Commitment Therapy processes

Authors: Ambra Mara Giovannetti, Jana Pöttgen, Elisenda Anglada, Rebeca Menendez, Jürgen Hoyer, Andrea Giordano, Kenneth Ian Pakenham, Ingrid Galán, Alessandra Solari

Corresponding author: Ambra Mara Giovannetti, ambra.giovannetti@istituto-besta.it
Fondazione IRCCS Istituto Neurologico Carlo Besta, Milan, Italy. Via Celoria 11, 20133 Milano, Italia

Supplementary File 3 "Spanish CompACT Translation grid"

COMMENTS/QUESTIONS MADE BY:
Please, use the appropriate colour based on this legenda.<br>[MFiF] _ _ _ (Consultant Spanish translator 1)<br>[AV] _ _ _ _(Consultant Spanish translator 2)<br>[EG] _ _ _ (Consultant backward translator)<br><br>[IG] _ _ _ (Researcher 1)<br>[EA] _ _ _ (Researcher2)<br>[RM]_ _ _ (Researcher 3)<br>[SF] _ _ _ (Lay person)<br>[CL] _ _ _ (ACT expert)<br><br>☞ query

TITLE

ORIGINAL ENGLISH WORDING	COMMENTS/QUESTIONS
CompACT (Comprehensive assessment of Acceptance and Commitment Therapy processes)	
**SPANISH TRANSLATION 1**	
CompACT (Comprehensive assessment of Acceptance and Commitment Therapy processes)	
**SPANISH TRANSLATION 2**	
CompACT (Comprehensive assessment of Acceptance and Commitment Therapy processes)	
**RECONCILED SPANISH TRANSLATION**	
CompACT (Comprehensive assessment of Acceptance and Commitment Therapy processes)	
**BACKWARD TRANSLATION**	
CompACT (Comprehensive assessment of Acceptance and Commitment Therapy processes)	
**ADVANCED SPANISH VERSION 1**	As for other PROMs, it was decided not to translate the title
CompACT (Comprehensive assessment of Acceptance and Commitment Therapy processes)	
**ADVANCED SPANISH VERSION 2 (post discussion with ACT expert)**	
CompACT (Comprehensive assessment of Acceptance and Commitment Therapy processes)	

FINAL SPANISH TRANSLATION	
CompACT (Comprehensive assessment of Acceptance and Commitment Therapy processes)	

INTRODUCTION

ORIGINAL ENGLISH WORDING	COMMENTS/QUESTIONS
Please rate the following 23 statements using the scale below:	
**SPANISH TRANSLATION 1**	
Por favor, puntúe las 23 afirmaciones que aparecen a continuación utilizando la siguiente escala:	
**SPANISH TRANSLATION 2**	
Por favor, evalúe las 23 frases siguientes mediante la escala que figura a continuación:	
**RECONCILED SPANISH TRANSLATION**	
Por favor, evalúe las 23 frases siguientes mediante la escala que figura a continuación:	
**BACKWARD TRANSLATION**	
Please evaluate the 23 sentences below using the following scale:	
**ADVANCED SPANISH VERSION 1**	Reconciled OK. No comments from the ACT expert.
Por favor, evalúe las 23 frases siguientes mediante la escala que figura a continuación:	
**ADVANCED SPANISH VERSION 2 (post discussion with ACT expert)**	
Por favor, evalúe las 23 frases siguientes mediante la escala que figura a continuación:	
**FINAL SPANISH TRANSLATION**	
Por favor, evalúe las 23 frases siguientes mediante la escala que figura a continuación:	

POSSIBLE REPLY # 1

ORIGINAL ENGLISH WORDING	COMMENTS/QUESTIONS
Strongly disagree	
**SPANISH TRANSLATION 1**	
Muy en desacuerdo	
**SPANISH TRANSLATION 2**	
Muy en desacuerdo	
**RECONCILED SPANISH TRANSLATION**	
Muy en desacuerdo	
**BACKWARD TRANSLATION**	
Disagree very much	

ADVANCED SPANISH VERSION 1	Reconciled OK. No comments from the ACT expert.
Muy en desacuerdo	
ADVANCED SPANISH VERSION 2 (post discussion with ACT expert)	
Muy en desacuerdo	
FINAL SPANISH TRANSLATION	
Muy en desacuerdo	

POSSIBLE REPLY # 2

ORIGINAL ENGLISH WORDING	COMMENTS/QUESTIONS
Moderately disagree	
SPANISH TRANSLATION 1	
Bastante en desacuerdo	
SPANISH TRANSLATION 2	
Bastante en desacuerdo	
RECONCILED SPANISH TRANSLATION	
Bastante en desacuerdo	
BACKWARD TRANSLATION	
Disagree quite a lot	
ADVANCED SPANISH VERSION 1	Reconciled OK. No comments from the ACT expert.
Bastante en desacuerdo	
ADVANCED SPANISH VERSION 2 (post discussion with ACT expert)	
Bastante en desacuerdo	
FINAL SPANISH TRANSLATION	
Bastante en desacuerdo	

POSSIBLE REPLY # 3

ORIGINAL ENGLISH WORDING	COMMENTS/QUESTIONS
Slightly disagree	
SPANISH TRANSLATION 1	
Algo en desacuerdo	
SPANISH TRANSLATION 2	
Poco en desacuerdo	
RECONCILED SPANISH TRANSLATION	
Algo en desacuerdo	

BACKWARD TRANSLATION	
Disagree somewhat	
ADVANCED SPANISH VERSION 1	Reconciled OK. No comments from the ACT expert.
Algo en desacuerdo	
ADVANCED SPANISH VERSION 2 (post discussion with ACT expert)	
Algo en desacuerdo	
FINAL SPANISH TRANSLATION	
Algo en desacuerdo	

POSSIBLE REPLY # 4

ORIGINAL ENGLISH WORDING	COMMENTS/QUESTIONS
Neither agree nor disagree	
SPANISH TRANSLATION 1	
Ni de acuerdo ni en desacuerdo	
SPANISH TRANSLATION 2	
Ni de acuerdo ni en desacuerdo	
RECONCILED SPANISH TRANSLATION	
Ni de acuerdo ni en desacuerdo	
BACKWARD TRANSLATION	
Neither agree or disagree	
ADVANCED SPANISH VERSION 1	Reconciled OK. No comments from the ACT expert.
Ni de acuerdo ni en desacuerdo	
ADVANCED SPANISH VERSION 2 (post discussion with ACT expert)	
Ni de acuerdo ni en desacuerdo	
FINAL SPANISH TRANSLATION	
Ni de acuerdo ni en desacuerdo	

POSSIBLE REPLY # 5

ORIGINAL ENGLISH WORDING	COMMENTS/QUESTIONS
Slightly agree	
SPANISH TRANSLATION 1	
Algo de acuerdo	
SPANISH TRANSLATION 2	

Poco de acuerdo	
**RECONCILED SPANISH TRANSLATION**	
Algo de acuerdo	
**BACKWARD TRANSLATION**	
Agree somewhat	
**ADVANCED SPANISH VERSION 1**	Reconciled OK. No comments from the ACT expert.
Algo de acuerdo	
**ADVANCED SPANISH VERSION 2 (post discussion with ACT expert)**	
Algo de acuerdo	
**FINAL SPANISH TRANSLATION**	
Algo de acuerdo	

POSSIBLE REPLY # 6

ORIGINAL ENGLISH WORDING	COMMENTS/QUESTIONS
Moderately agree	
**SPANISH TRANSLATION 1**	
Bastante de acuerdo	
**SPANISH TRANSLATION 2**	
Bastante de acuerdo	
**RECONCILED SPANISH TRANSLATION**	
Bastante de acuerdo	
**BACKWARD TRANSLATION**	
Agree quite a lot	
**ADVANCED SPANISH VERSION 1**	Reconciled OK. No comments from the ACT expert.
Bastante de acuerdo	
**ADVANCED SPANISH VERSION 2 (post discussion with ACT expert)**	
Bastante de acuerdo	
**FINAL SPANISH TRANSLATION**	
Bastante de acuerdo	

POSSIBLE REPLY # 7

ORIGINAL ENGLISH WORDING	COMMENTS/QUESTIONS
Strongly agree	
**SPANISH TRANSLATION 1**	

Muy de acuerdo	
**SPANISH TRANSLATION 2**	
Muy de acuerdo	
**RECONCILED SPANISH TRANSLATION**	
Muy de acuerdo	
**BACKWARD TRANSLATION**	
Agree very much	
**ADVANCED SPANISH VERSION 1**	Reconciled OK. No comments from the ACT expert.
Muy de acuerdo	
**ADVANCED SPANISH VERSION 2 (post discussion with ACT expert)**	
Muy de acuerdo	
**FINAL SPANISH TRANSLATION**	
Muy de acuerdo	

ITEM # 1

ORIGINAL ENGLISH WORDING	COMMENTS/QUESTIONS
I can identify the things that really matter to me in life and pursue them	
**SPANISH TRANSLATION 1**	
1. Soy capaz de identificar las cosas que cuentan de verdad para mí en la vida y de ir a por ellas.	
**SPANISH TRANSLATION 2**	
1. Puedo decir qué cosas me importan de verdad en la vida y dedicarme a ellas	
**RECONCILED SPANISH TRANSLATION**	
1. Soy capaz de decir qué cosas me importan de verdad en la vida y dedicarme a ellas.	
**BACKWARD TRANSLATION**	
1. I am able to say which things are really important to me in life and concentrate on them	
**ADVANCED SPANISH VERSION 1**	
1. Soy capaz de decir qué cosas me importan de verdad en la vida y dedicarme a ellas.	
**ADVANCED SPANISH VERSION 2 (post discussion with ACT expert)**	
1. Soy capaz de decir qué cosas me importan de verdad en la vida y dedicarme a ellas.	
**FINAL SPANISH TRANSLATION**	
1. Soy capaz de decir qué cosas me importan de verdad en la vida y dedicarme a ellas.	

ITEM # 2

ORIGINAL ENGLISH WORDING	COMMENTS/QUESTIONS
One of my big goals is to be free from painful emotions	
**SPANISH TRANSLATION 1**	
2. Uno de mis grandes objetivos es vivir sin emociones dolorosas.	
**SPANISH TRANSLATION 2**	
2. Una de mis grandes metas es no tener emociones dolorosas	
**RECONCILED SPANISH TRANSLATION**	
2. Uno de mis grandes objetivos es vivir sin emociones dolorosas.	
**BACKWARD TRANSLATION**	
2. One of my big goals is to live without painful emotions	
**ADVANCED SPANISH VERSION 1**	Reconciled OK. No comments from the ACT expert.
2. Uno de mis grandes objetivos es vivir sin emociones dolorosas.	
**ADVANCED SPANISH VERSION 2 (post discussion with ACT expert)**	
2. Uno de mis grandes objetivos es vivir sin emociones dolorosas.	
**FINAL SPANISH TRANSLATION**	
2. Uno de mis grandes objetivos es vivir sin emociones dolorosas.	

ITEM # 3

ORIGINAL ENGLISH WORDING	COMMENTS/QUESTIONS
I rush through meaningful activities without being really attentive to them	
**SPANISH TRANSLATION 1**	
3. Me apresuro al hacer actividades que tienen sentido para mí, sin prestarles verdadera atención.	
**SPANISH TRANSLATION 2**	
3. Hago a toda prisa actividades importantes sin prestarles realmente atención	
**RECONCILED SPANISH TRANSLATION**	
3. Hago a toda prisa actividades importantes para mí sin prestarles verdadera atención	
**BACKWARD TRANSLATION**	
3. I rush through activities that are important to me without paying real attention to them	
**ADVANCED SPANISH VERSION 1**	Reconciled OK. No comments from the ACT expert.
3. Hago a toda prisa actividades importantes para mí sin prestarles verdadera atención	
**ADVANCED SPANISH VERSION 2 (post discussion with ACT expert)**	
3. Hago a toda prisa actividades importantes para mí sin prestarles verdadera atención	

FINAL SPANISH TRANSLATION	
3. Hago a toda prisa actividades importantes para mí sin prestarles verdadera atención	

ITEM # 4

ORIGINAL ENGLISH WORDING	COMMENTS/QUESTIONS
I try to stay busy to keep thoughts or feelings from coming	
**SPANISH TRANSLATION 1**	
4. Intento mantenerme ocupado/a para evitar que me vengan pensamientos o sentimientos	
**SPANISH TRANSLATION 2**	
4. Intento mantenerme ocupado/a para que no me vengan ideas o sentimientos a la cabeza	
**RECONCILED SPANISH TRANSLATION**	
4. Intento mantenerme ocupado/a para evitar que me vengan pensamientos o sentimientos	
**BACKWARD TRANSLATION**	
4. I try to keep myself busy to avoid thoughts or feelings coming up on me	
**ADVANCED SPANISH VERSION 1**	Reconciled OK. No comments from the ACT expert.
4. Intento mantenerme ocupado/a para evitar que me vengan pensamientos o sentimientos	
**ADVANCED SPANISH VERSION 2 (post discussion with ACT expert)**	
4. Intento mantenerme ocupado/a para evitar que me vengan pensamientos o sentimientos	
**FINAL SPANISH TRANSLATION**	
4. Intento mantenerme ocupado/a para evitar que me vengan pensamientos o sentimientos	

ITEM # 5

ORIGINAL ENGLISH WORDING	COMMENTS/QUESTIONS
I act in ways that are consistent with how I wish to live my life	
**SPANISH TRANSLATION 1**	
5. Actúo de forma coherente con la manera en que deseo vivir mi vida.	
**SPANISH TRANSLATION 2**	
5. Mis maneras de actuar concuerdan con la forma en que deseo vivir la vida	
**RECONCILED SPANISH TRANSLATION**	
5. Mis maneras de actuar concuerdan con la forma en que deseo vivir la vida	
**BACKWARD TRANSLATION**	
5. The ways I act match how I wish to live life	
**ADVANCED SPANISH VERSION 1**	Reconciled OK. No comments from the ACT expert.

5. Mis maneras de actuar concuerdan con la forma en que deseo vivir la vida	
**ADVANCED SPANISH VERSION 2 (post discussion with ACT expert)**	
5. Mis maneras de actuar concuerdan con la forma en que deseo vivir la vida	
**FINAL SPANISH TRANSLATION**	
5. Mis maneras de actuar concuerdan con la forma en que deseo vivir la vida	

ITEM # 6

ORIGINAL ENGLISH WORDING	COMMENTS/QUESTIONS
I get so caught up in my thoughts that I am unable to do the things that I most want to do	
**SPANISH TRANSLATION 1**	
6. Me quedo tan enfrascado/a en mis pensamientos que soy incapaz de hacer las cosas que más quiero hacer.	
**SPANISH TRANSLATION 2**	
6. Me quedo tan absorto/a en mis pensamientos que no soy capaz de hacer las cosas que más deseo	
**RECONCILED SPANISH TRANSLATION**	We have the doubt that "enfrascado/a" is not easy to be understood.
6. Me quedo tan enfrascado/a en mis pensamientos que soy incapaz de hacer las cosas que más deseo	
**BACKWARD TRANSLATION**	
6. I get so stuck in my thoughts that I'm unable to do the things that I most wish to do	
**ADVANCED SPANISH VERSION 1**	Reconciled OK.<br>I suggest to use "absorto" instead of "enfrascado"
6. Me quedo tan enfrascado en mis pensamientos que soy incapaz de hacer las cosas que más deseo	
**ADVANCED SPANISH VERSION 2 (post discussion with ACT expert)**	
6. Me quedo tan absorto/a en mis pensamientos que soy incapaz de hacer las cosas que más deseo.	
**FINAL SPANISH TRANSLATION**	
6. Me quedo tan absorto/a en mis pensamientos que soy incapaz de hacer las cosas que más deseo.	

ITEM # 7

ORIGINAL ENGLISH WORDING	COMMENTS/QUESTIONS
I make choices based on what is important to me, even if it is stressful	
**SPANISH TRANSLATION 1**	
7. Decido las cosas en función de lo que para mí es importante, aunque resulte estresante.	

**SPANISH TRANSLATION 2**	
7. Tomo decisiones en función de lo que es importante para mí, aunque resulte estresante	
**RECONCILED SPANISH TRANSLATION**	
7. Tomo decisiones en función de lo que es importante para mí, aunque resulte estresante	
**BACKWARD TRANSLATION**	
7. I make decisions according to what is important to me, even if it's stressful	
**ADVANCED SPANISH VERSION 1**	I suggest to use “Elijo hacer lo que es importante” instead of “Tomo decisiones en función de lo que es importante”. Reconciled OK. The issue is about the meaning of the action: I decided (just in my mind“) or think about something and take an action.
7. Tomo decisiones en función de lo que es importante para mí, aunque resulte estresante	
**ADVANCED SPANISH VERSION 2 (post discussion with ACT expert)**	
7. Elijo hacer lo que es importante para mí, aunque resulte estresante	
**FINAL SPANISH TRANSLATION**	
7. Elijo hacer lo que es importante para mí, aunque resulte estresante	

ITEM # 8

**ADVANCED SPANISH VERSION 1**	**COMMENTS/QUESTIONS**
I tell myself that I shouldn’t have certain thoughts	
**SPANISH TRANSLATION 1**	
8. Me digo a mí mismo/a que no debería tener determinados pensamientos	
**SPANISH TRANSLATION 2**	
8. Me digo a mí mismo/a que no debiera tener ciertos pensamientos	
**RECONCILED SPANISH TRANSLATION**	The question remains whether "certain thoughts" is understood as a euphemism.
8. Me digo a mí mismo/a que no debería tener determinados pensamientos	
**BACKWARD TRANSLATION**	
8. I tell myself that I shouldn't have certain thoughts	
**ADVANCED SPANISH VERSION 1**	Reconciled OK. I suggest to use “determinados” instead of “ciertos”
8. Me digo a mí mismo/a que no debería tener determinados pensamientos	
**ADVANCED SPANISH VERSION 2 (post discussion with ACT expert)**	
8.Me digo a mí mismo/a que no debería tener ciertos pensamientos.	
**FINAL SPANISH TRANSLATION**	
8.Me digo a mí mismo/a que no debería tener ciertos pensamientos.	

ITEM # 9

ORIGINAL ENGLISH WORDING	COMMENTS/QUESTIONS
I find it difficult to stay focused on what's happening in the present	
**SPANISH TRANSLATION 1**	
9. Me resulta difícil mantenerme centrado/a en lo que está sucediendo en el presente.	
**SPANISH TRANSLATION 2**	
9. Tengo dificultades para mantenerme centrado/a en lo que sucede en el presente	
**RECONCILED SPANISH TRANSLATION**	
9. Me resulta difícil mantenerme centrado/a en lo que sucede en el presente	
**BACKWARD TRANSLATION**	
9. I find it difficult to keep focused on what is happening in the present	
**ADVANCED SPANISH VERSION 1**	Reconciled OK. No comments from the ACT expert.
9. Me resulta difícil mantenerme centrado/a en lo que sucede en el presente	
**ADVANCED SPANISH VERSION 2 (post discussion with ACT expert)**	
9. Me resulta difícil mantenerme centrado/a en lo que sucede en el presente	
**FINAL SPANISH TRANSLATION**	
9. Me resulta difícil mantenerme centrado/a en lo que sucede en el presente	

ITEM # 10

ORIGINAL ENGLISH WORDING	COMMENTS/QUESTIONS
I behave in line with my personal values	
**SPANISH TRANSLATION 1**	
10. Me comporto de forma acorde con mis valores personales.	
**SPANISH TRANSLATION 2**	
10. Me comporto con arreglo a mis valores personales	
**RECONCILED SPANISH TRANSLATION**	
10. Me comporto de forma acorde con mis valores personales	
**BACKWARD TRANSLATION**	
10. I behave in a way that reflects my personal values	
**ADVANCED SPANISH VERSION 1**	Reconciled OK.<br>I suggest to use "fiel", "de forma fiel" instead of "acorde"
10. Me comporto de forma acorde con mis valores personales	
**ADVANCED SPANISH VERSION 2 (post discussion with ACT expert)**	
10. Me comporto de forma fiel a mis valores personales	
**FINAL SPANISH TRANSLATION**	

10. Me comporto de forma fiel a mis valores personales	

ITEM # 11

ORIGINAL ENGLISH WORDING	COMMENTS/QUESTIONS
I go out of my way to avoid situations that might bring difficult thoughts, feelings, or sensations	
**SPANISH TRANSLATION 1**	
11. Hago todo lo que puedo para evitar situaciones que puedan traerme pensamientos, sentimientos o sensaciones difíciles.	
**SPANISH TRANSLATION 2**	
11. Me aparto de mi ruta para evitar situaciones que puedan traerme ideas, sentimientos o sensaciones difíciles	
**RECONCILED SPANISH TRANSLATION**	
11. Hago todo lo que puedo para evitar situaciones que puedan traerme pensamientos, sentimientos o sensaciones difíciles.	
**BACKWARD TRANSLATION**	
11. I do everything I can to avoid situations that might bring me difficult thoughts, feelings or sensations	
**ADVANCED SPANISH VERSION 1**	Reconciled OK. No comments from the ACT expert.
11. Me esfuerzo por evitar situaciones que puedan traerme pensamientos, sentimientos o sensaciones difíciles.	
**ADVANCED SPANISH VERSION 2 (post discussion with ACT expert)**	
11. Me esfuerzo por evitar situaciones que puedan traerme pensamientos, sentimientos o sensaciones difíciles.	
**FINAL SPANISH TRANSLATION**	
11. Me esfuerzo por evitar situaciones que puedan traerme pensamientos, sentimientos o sensaciones difíciles.	

ITEM # 12

ORIGINAL ENGLISH WORDING	COMMENTS/QUESTIONS
Even when doing the things that matter to me, I find myself doing them without paying attention	
**SPANISH TRANSLATION 1**	
12. Incluso cuando hago las cosas que me importan, descubro que las estoy haciendo sin prestar atención	

**SPANISH TRANSLATION 2**	
12. Incluso cuando hago cosas que me interesan, veo que las hago sin prestar atención	
**RECONCILED SPANISH TRANSLATION**	
12. Incluso cuando hago las cosas que me importan, veo que las hago sin prestar atención	
**BACKWARD TRANSLATION**	
12. Even when I do things that are important to me, I see that I do them without paying attention	
**ADVANCED SPANISH VERSION 1**	Reconciled OK. No comments from the ACT expert.
12. Incluso cuando estoy haciendo las cosas que me importan, veo que las estoy haciendo sin prestar atención	
**ADVANCED SPANISH VERSION 2 (post discussion with ACT expert)**	
12. Incluso cuando estoy haciendo las cosas que me importan, veo que las estoy haciendo sin prestar atención	
**FINAL SPANISH TRANSLATION**	
12. Incluso cuando estoy haciendo las cosas que me importan, veo que las estoy haciendo sin prestar atención	

ITEM # 13

ORIGINAL ENGLISH WORDING	COMMENTS/QUESTIONS
I am willing to fully experience whatever thoughts, feelings and sensations come up for me, without trying to change or defend against them	
**SPANISH TRANSLATION 1**	
13. Estoy dispuesto/a a experimentar plenamente cualesquiera pensamientos, sentimientos y sensaciones que me puedan surgir, sin intentar cambiarlos ni defenderme ante ellos.	
**SPANISH TRANSLATION 2**	
13. Estoy dispuesto/a a vivir plenamente todas las ideas, sentimientos o sensaciones que me surjan, sin intentar cambiarlas ni blindarme ante ellas	
**RECONCILED SPANISH TRANSLATION**	
13. Estoy dispuesto/a a vivir plenamente todos los pensamientos, sentimientos y sensaciones que me surjan, sin intentar cambiarlos ni defenderme de ellos	
**BACKWARD TRANSLATION**	
13. I am willing to fully live all the thoughts, feelings and sensations that arise in me, without trying to change them or defend myself from them	
**ADVANCED SPANISH VERSION 1**	Reconciled OK.<br>The important concept here is that I am willing to fully FEEL all
13. Estoy dispuesto/a a vivir plenamente todos los pensamientos, sentimientos y	

sensaciones que<br>me surjan, sin intentar cambiarlos ni defenderme de ellos	the thoughts, feelings and sensations that arise, without trying to change them or FIGHT AGAINST them I suggest you to use "sentir" and "pelear"
**ADVANCED SPANISH VERSION 2 (post discussion with ACT expert)**	
13. Estoy dispuesto/a a sentir plenamente todos los pensamientos, sentimientos y sensaciones que me surjan, sin intentar cambiarlos ni pelear contra ellos.	
**FINAL SPANISH TRANSLATION**	
13. Estoy dispuesto/a a sentir plenamente todos los pensamientos, sentimientos y sensaciones que me surjan, sin intentar cambiarlos ni pelear contra ellos.	

ITEM # 14

ORIGINAL ENGLISH WORDING	COMMENTS/QUESTIONS
I undertake things that are meaningful to me, even when I find it hard to do so	
**SPANISH TRANSLATION 1**	
14. Emprendo cosas que para mí tienen sentido, incluso cuando me resulta difícil hacerlo.	
**SPANISH TRANSLATION 2**	
14. Emprendo cosas que considero importantes, incluso cuando me resulta difícil hacerlo	
**RECONCILED SPANISH TRANSLATION**	
14. Empiezo cosas que son importantes para mí incluso cuando me resulta difícil hacerlo	
**BACKWARD TRANSLATION**	
14. I start things that are important to me even when it's hard for me to do so	
**ADVANCED SPANISH VERSION 1**	Reconciled OK.<br>I suggest to use LLEVO A CABO instead of Empiezo
14. Empiezo cosas que son importantes para mí incluso cuando me resulta difícil hacerlo	
**ADVANCED SPANISH VERSION 2 (post discussion with ACT expert)**	
14. Llevo a cabo cosas que son importantes para mí incluso cuando me resulta difícil hacerlo.	
**FINAL SPANISH TRANSLATION**	
14. Llevo a cabo cosas que son importantes para mí incluso cuando me resulta difícil hacerlo.	

ITEM # 15

ORIGINAL ENGLISH WORDING	COMMENTS/QUESTIONS
I work hard to keep out upsetting feelings	
**SPANISH TRANSLATION 1**	

15. Me esfuerzo mucho por apartar los sentimientos que me puedan alterar.	
**SPANISH TRANSLATION 2**	
15. Me esfuerzo mucho por mantener a raya sentimientos que me causen malestar	
**RECONCILED SPANISH TRANSLATION**	
15. Me esfuerzo mucho por apartar los sentimientos que me puedan alterar.	
**BACKWARD TRANSLATION**	
15. I make a big effort to put aside feelings that might upset me	
**ADVANCED SPANISH VERSION 1**	Reconciled OK. No comments from the ACT expert.
15. Me esfuerzo mucho por apartar los sentimientos que me puedan alterar.	
**ADVANCED SPANISH VERSION 2 (post discussion with ACT expert)**	
15. Me esfuerzo mucho por apartar los sentimientos que me puedan alterar.	
**FINAL SPANISH TRANSLATION**	
15. Me esfuerzo mucho por apartar los sentimientos que me puedan alterar.	

ITEM # 16

**ORIGINAL ENGLISH WORDING**	**COMMENTS/QUESTIONS**
I do jobs or tasks automatically, without being aware of what I'm doing	
**SPANISH TRANSLATION 1**	
16. Llevo a cabo trabajos o tareas de forma automática, sin darme cuenta de lo que estoy haciendo.	
**SPANISH TRANSLATION 2**	
16. Hago trabajos o tareas mecánicamente, sin conciencia de lo que estoy haciendo	
**RECONCILED SPANISH TRANSLATION**	
16. Hago trabajos o tareas mecánicamente, sin darme cuenta de lo que estoy haciendo.	
**BACKWARD TRANSLATION**	
16. I do jobs or tasks mechanically, without realizing what I'm doing	
**ADVANCED SPANISH VERSION 1**	Reconciled OK. No comments from the ACT expert.
16. Hago trabajos o tareas mecánicamente, sin darme cuenta de lo que estoy haciendo.	
**ADVANCED SPANISH VERSION 2 (post discussion with ACT expert)**	
16. Hago trabajos o tareas mecánicamente, sin darme cuenta de lo que estoy haciendo.	
**FINAL SPANISH TRANSLATION**	
16. Hago trabajos o tareas mecánicamente, sin darme cuenta de lo que estoy haciendo.	

ITEM # 17

ORIGINAL ENGLISH WORDING	COMMENTS/QUESTIONS
I am able to follow my long terms plans including times when progress is slow	
**SPANISH TRANSLATION 1**	
17. Consigo atenerme a mis planes a largo plazo, incluso en los periodos en que los progresos son lentos.	
**SPANISH TRANSLATION 2**	
17. Soy capaz de atenerme a mis planes de largo plazo aunque a veces avance despacio	
**RECONCILED SPANISH TRANSLATION**	
17. Soy capaz de seguir mis planes a largo plazo incluso cuando avanzo despacio	
**BACKWARD TRANSLATION**	
17. I am able to follow my long-term plans even when I make slow progress	
**ADVANCED SPANISH VERSION 1**	Reconciled OK. No comments from the ACT expert.
17. Soy capaz de seguir mis planes a largo plazo incluso cuando avanzan despacio	
**ADVANCED SPANISH VERSION 2 (post discussion with ACT expert)**	
17. Soy capaz de seguir mis planes a largo plazo incluso cuando avanzan despacio	
**FINAL SPANISH TRANSLATION**	
17. Soy capaz de seguir mis planes a largo plazo incluso cuando avanzan despacio	

ITEM # 18

ORIGINAL ENGLISH WORDING	COMMENTS/QUESTIONS
Even when something is important to me, I'll rarely do it if there is a chance it will upset me	
**SPANISH TRANSLATION 1**	
18. Incluso si hay algo que me importa mucho, rara vez lo hago si existe la posibilidad de que me altere.	
**SPANISH TRANSLATION 2**	
18. Aunque algo me resulte importante, rara vez lo hago si existe la posibilidad de que me moleste	
**RECONCILED SPANISH TRANSLATION**	
18. Aunque algo me resulte importante, rara vez lo hago si existe la posibilidad de que me altere	
**BACKWARD TRANSLATION**	
18. Even if something is important to me, I rarely do it if there is a chance of it upsetting me	
**ADVANCED SPANISH VERSION 1**	Reconciled OK. No comments from the ACT expert.
18. Aunque algo me resulte importante, rara vez lo hago si existe la posibilidad de que me	

altere	
**ADVANCED SPANISH VERSION 2 (post discussion with ACT expert)**	
18. Aunque algo me resulte importante, rara vez lo hago si existe la posibilidad de que me altere	
**FINAL SPANISH TRANSLATION**	
18. Aunque algo me resulte importante, rara vez lo hago si existe la posibilidad de que me altere	

ITEM # 19

ORIGINAL ENGLISH WORDING	COMMENTS/QUESTIONS
It seems I am "running on automatic" without much awareness of what I'm doing	
**SPANISH TRANSLATION 1**	
19. Parece como si fuera "con piloto automático" sin fijarme mucho en lo que estoy haciendo.	
**SPANISH TRANSLATION 2**	
19. Parece que «voy con el piloto automático» sin mayor conciencia de lo que hago	
**RECONCILED SPANISH TRANSLATION**	
19. Parece que «voy con el piloto automático» sin fijarme mucho en lo que estoy haciendo.	
**BACKWARD TRANSLATION**	
19. It seems I'm on "automatic pilot", not noticing much what I'm doing	
**ADVANCED SPANISH VERSION 1**	Reconciled OK. No comments from the ACT expert.
19. Parece que «voy con el piloto automático» sin fijarme mucho en lo que estoy haciendo.	
**ADVANCED SPANISH VERSION 2 (post discussion with ACT expert)**	
19. Parece que «voy con el piloto automático» sin fijarme mucho en lo que estoy haciendo.	
**FINAL SPANISH TRANSLATION**	
19. Parece que «voy con el piloto automático» sin fijarme mucho en lo que estoy haciendo.	

ITEM # 20

ORIGINAL ENGLISH WORDING	COMMENTS/QUESTIONS
Thoughts are just thoughts – they don't control what I do	
**SPANISH TRANSLATION 1**	
20. Los pensamientos son solo pensamientos: no controlan lo que hago.	
**SPANISH TRANSLATION 2**	
20. Las ideas son solo eso, ideas, y no controlan lo que hago	
**RECONCILED SPANISH TRANSLATION**	

20. Los pensamientos son solo pensamientos y no controlan lo que hago	
**BACKWARD TRANSLATION**	
20.Thoughts are just thoughts and they don't control what I do	
**ADVANCED SPANISH VERSION 1**	Reconciled OK. No comments from the ACT expert.
20. Los pensamientos son solo pensamientos y no controlan lo que hago	
**ADVANCED SPANISH VERSION 2 (post discussion with ACT expert)**	
20. Los pensamientos son solo pensamientos y no controlan lo que hago	
**FINAL SPANISH TRANSLATION**	
20. Los pensamientos son solo pensamientos y no controlan lo que hago	

ITEM # 21

ORIGINAL ENGLISH WORDING	COMMENTS/QUESTIONS
My values are really reflected in my behaviour	
**SPANISH TRANSLATION 1**	
21. Mis valores se ven fielmente reflejados en mi comportamiento.	
**SPANISH TRANSLATION 2**	
21. Mis valores se reflejan bien en mi comportamiento	
**RECONCILED SPANISH TRANSLATION**	
21. Mis valores se reflejan fielmente en mi comportamiento	
**BACKWARD TRANSLATION**	
21.My values are faithfully reflected in my behavior	
**ADVANCED SPANISH VERSION 1**	Reconciled OK. No comments from the ACT expert.
21. Mis valores se reflejan fielmente en mi comportamiento	
**ADVANCED SPANISH VERSION 2 (post discussion with ACT expert)**	
21. Mis valores se reflejan fielmente en mi comportamiento	
**FINAL SPANISH TRANSLATION**	
21. Mis valores se reflejan fielmente en mi comportamiento	

ITEM # 22

ORIGINAL ENGLISH WORDING	COMMENTS/QUESTIONS
I can take thoughts and feelings as they come, without attempting to control or avoid them	
**SPANISH TRANSLATION 1**	
22. Soy capaz de aceptar los pensamientos y sentimientos tal como llegan, sin intentar controlarlos ni evitarlos.	

SPANISH TRANSLATION 2	
22. Puedo encajar las ideas y los sentimientos tal cual llegan, sin intentar controlarlos ni evitarlos	
**RECONCILED SPANISH TRANSLATION**	
22. Soy capaz de aceptar los pensamientos y sentimientos tal como llegan, sin intentar controlarlos ni evitarlos	
**BACKWARD TRANSLATION**	
22. I am able to accept thoughts and feelings as they appear, without trying to control them or avoid them	
**ADVANCED SPANISH VERSION 1**	Reconciled OK. No comments from the ACT expert.
22. Soy capaz de aceptar los pensamientos y sentimientos tal como llegan, sin intentar controlarlos<br>ni evitarlos	
**ADVANCED SPANISH VERSION 2 (post discussion with ACT expert)**	
22. Soy capaz de aceptar los pensamientos y sentimientos tal como llegan, sin intentar controlarlos<br>ni evitarlos	
**FINAL SPANISH TRANSLATION**	
22. Soy capaz de aceptar los pensamientos y sentimientos tal como llegan, sin intentar controlarlos<br>ni evitarlos	

ITEM # 23

ORIGINAL ENGLISH WORDING	COMMENTS/QUESTIONS
I can keep going with something when it's important to me	
**SPANISH TRANSLATION 1**	
23. Soy capaz de seguir adelante con las cosas que me importan.	
**SPANISH TRANSLATION 2**	
23. Puedo seguir adelante con algo cuando algo me resulta importante	
**RECONCILED SPANISH TRANSLATION**	
23. Soy capaz de seguir adelante con las cosas cuando me importan	
**BACKWARD TRANSLATION**	
23. I am able to get on with things when they are important to me	
**ADVANCED SPANISH VERSION 1**	Reconciled OK. No comments from the ACT expert.
23. Soy capaz de seguir adelante con las cosas cuando me importan	
**ADVANCED SPANISH VERSION 2 (post discussion with ACT expert)**	

23. Soy capaz de seguir adelante con las cosas cuando me importan	
**FINAL SPANISH TRANSLATION**	
23. Soy capaz de seguir adelante con las cosas cuando me importan	
