# Supplementary material for: Cross-Country Adaptation of a Psychological Flexibility Measure: The Comprehensive Assessment of Acceptance and Commitment Therapy Processes
Source: Int J Environ Res Public Health. 2022 Mar 8;19(6):3150. doi: 10.3390/ijerph19063150 (PMC8953951; doi:10.3390/ijerph19063150)
Supplement: Supplementary file 1 [file ijerph-19-03150-s001.zip › Supplementary File 4 - Content Analysis Quotes.pdf]

Article title: Cross-country adaptation of a psychological flexibility measure: The Comprehensive assessment of Acceptance and Commitment Therapy processes

Authors: Ambra Mara Giovannetti, Jana Pöttgen, Elisenda Anglada, Rebeca Menendez, Jürgen Hoyer, Andrea Giordano, Kenneth Ian Pakenham, Ingrid Galán, Alessandra Solari

Corresponding author: Ambra Mara Giovannetti, ambra.giovannetti@istituto-besta.it
Fondazione IRCCS Istituto Neurologico Carlo Besta, Milan, Italy. Via Celoria 11, 20133 Milano, Italia

Supplementary File 4 – Audit trail

Cognitive Debriefing Guide

- Provide verbal information on the study objective
- Gather
  - the signed informed consent form
  - the completed general questionnaire
  - The sociodemographic information (Age, gender, education)
- Ask the participant to complete the CompACT, on his/her own.
- Start the interview, taking written notes of participant's reply.

General question

- Did you find it hard to complete the questionnaire? If yes, why?

  <u>Prompts (if useful):</u>
  - What do you think about questionnaire's length, layout, and readability?

Introduction

- Could you please explain to me, in your own words, what this statement means?

  <u>Prompts (if useful):</u>
  - Did you find unclear words or expressions?

Items

Please, consider each item one after the other and pose the following questions when pertinent:

- What does this statement mean?

  <u>Prompts (if useful):</u>
  - Could you please rephrase it in your own words?
  - Are there any unclear words?
  - Did you find it hard to answer this question? If yes, why?

Response options

- Did you find it hard to rate the items? If yes, why?

Do you have any other comments?

Content Analysis Quotes

Where in the questionnaire	Content of concern	Quotes
Item 2 - One of my big goals is to be free from painful emotions	- *Item too vague*<br><br><br>- *Unclear expression: "big goal"*	- *This item is not enough specific (G6, PwMS)*<br>- *I think it is too vague, I am not sure what is focused on (I9, PwMS)*<br>- *Content of this question is not clear- what is a "big goal"? A life goal? (G5, PwMS)*
Item 3 - I rush through meaningful activities without being really attentive to them	- *Unclear item*<br><br><br>- *Unclear expression: "meaningful activities"*	- *I had to read it twice. It is hard to explain it in my own words. I would delete the expression "a toda prisa" ["rush" in the English version] (S2, GP)*<br>- *What exactly does "meaningful activities refer to (e.g. job, social environment, activities in general)? (G2, GP)*
Item 4 - I try to stay busy to keep thoughts or feelings from coming	- *Unclear item*<br><br><br>- *Unclear expressions: "thoughts" and "feelings"*	- *I found it unclear (G2, GP)*<br><br><br>- *"There is no definition of which kind of thoughts or feelings. I cannot understand." (I1, GP)*<br>- *"Do you mean feelings in general or certain feelings?" (I3, GP)*
Item 5 - I act in ways that are consistent with how I wish to live my life	- *Item too vague*	- *This item is not enough specific (G6, PwMS)*
Item 6 - I get so caught up in my thoughts that I am unable to do the things that I most want to do	- *Unclear item, too nested*	- *This question is more complicated because it is very nested (G1, GP)*<br>- *Very difficult to understand (S4, GP)*

Item 13 - I am willing to fully experience whatever thoughts, feelings and sensations come up for me, without trying to change or defend against them	- *Unclear expression: “to fully experience”.*	- *“I cannot translate “to fully experience” in something concrete. I need an example. What should I notice in my behavior to answer this question?” (I1, GP)*
Item 20 - Thoughts are just thoughts – they don’t control what I do	- *Unclear item*	- *"It's hard to understand at first. I had to read it twice“ (S1, GP)*<br>- *“I found it really difficult, You can reply only if your self-awareness is high” (I1, GP)*
	- *Unclear expression: “thoughts do not control what I do”*	- *It is unclear to me what does it mean the expression “thoughts do not control what I do”. I think an example may be useful (I8, PwMS)*
	- *Unclear expression: “thoughts are just thoughts”*	- *“What does it mean “Thoughts are just thoughts”? I would suggest to modify the item as follow: “Capisco che I pensieri su di me non sono sempre oggettivi e non permetto loro di condizionare quello che faccio”. (I4, GP)*<br>- *“It is too abstract and vague. I found the expression “thoughts are just thoughts” denigrating towards the person who has this thought. Do you mean: Thoughts do not impede me to do what I do in daily life? Are you saying they are not useful?” (I1, GP)*<br>- *“thoughts are just thoughts”, so what? What is the implication? (I9, PwMS)*
Item 22 - I can take thoughts and feelings as they come, without attempting to control or avoid them	- *Unclear expression: “take thoughts and feeling as they come”.*	- *“I cannot translate “take thoughts and feeling as they come” in something concrete. I need an example. What should I notice in my behavior to answer this question?” (I1, GP)*
Response options	- *Difficult to understand the response gradient*	- *I experienced some difficulties with the response gradient. I found it difficult to understand the differences (S7, PwMS)*<br>- *I had problems understanding the scoring system (S8, PwMS)*

		- *Some items are not easy to understand, it is difficult to choose the option (S4, GP)*
	- *Option 3 ("Neither agree nor disagree") is not useful*	- *I think it is better not to give the option "Neither agree nor disagree". The person should decide what to answer. I would delete this response option (I12, PwMS)*
	- *Use number instead of label*	- *My suggestion is to use only number instead of number and labels (S1, GP)*
General comments	- *Too long statements (items)*	- *I found questions too long (S8, PwMS)*<br>- *Aren't these statements too long? (S9, PwMS)*
	- *Unclear item, too nested*	- *Basically not complicated, but the first question was irritating. That was two questions at once, so it was difficult to decide on an answer (G3, GP)*<br>- *Some items are not easy to understand, it is difficult to choose the option and it seems that some are repeated (S4, GP)*
	- *Concerns on possible comprehension difficulties for people with cognitive impairment*	- *Some questions are very long. I was wondering if there could be comprehension problems for patients with cognitive impairment (S8, PwMS)*<br>- *Due to questions length, patients with cognitive impairments may experience some difficulties (S5, PwMS)*
	- *Identical items*	- *Item 16 and 19 are really similar (G6, PwMS)*<br>- *These two items [13 and 22]are identical (I7, PwMS)*<br>- *Item 13 and 22 asked the same think (I9, PwMS)*<br>- *Some items are not easy to understand, it is difficult to choose the option and it seems that some are repeated (S4, GP)*<br>- *"22 is identical to 13" (I1, GP)*
	- *Include examples to clarify items*	- *Adding an example could be useful for reducing item abstractness (I1, GP)*
