# Supplementary material for: Cross-Country Adaptation of a Psychological Flexibility Measure: The Comprehensive Assessment of Acceptance and Commitment Therapy Processes
Source: Int J Environ Res Public Health. 2022 Mar 8;19(6):3150. doi: 10.3390/ijerph19063150 (PMC8953951; doi:10.3390/ijerph19063150)
Supplement: Supplementary file 1 [file ijerph-19-03150-s001.zip › Supplementary File 5 - CompACT versions.pdf]

Article title: Cross-country adaptation of a psychological flexibility measure: The Comprehensive assessment of Acceptance and Commitment Therapy processes

Authors: Ambra Mara Giovannetti, Jana Pöttgen, Elisenda Anglada, Rebeca Menendez, Jürgen Hoyer, Andrea Giordano, Kenneth Ian Pakenham, Ingrid Galán, Alessandra Solari

Corresponding author: Ambra Mara Giovannetti, ambra.giovannetti@istituto-besta.it
Fondazione IRCCS Istituto Neurologico Carlo Besta, Milan, Italy. Via Celoria 11, 20133 Milano, Italia

Supplementary File 5 – CompACT translation in Italian, German and Spanish

Italian CompACT

Codice ID:	Data:

Per ciascuna delle 23 affermazioni che seguono, indichi con una crocetta il suo grado di accordo/disaccordo

0	1	2	3	4	5	6
Totalmente in disaccordo	Moderatamente in disaccordo	Leggermente in disaccordo	Né d'accordo né in disaccordo	Leggermente d'accordo	Moderatamente d'accordo	Totalmente d'accordo

1. Sono in grado di riconoscere le cose che sono veramente importanti per me nella vita e di portarle avanti	0	1	2	3	4	5	6
2. Uno dei miei obiettivi principali è quello di non avere emozioni dolorose	0	1	2	3	4	5	6
3. Svolgo frettolosamente attività importanti, senza in realtà prestare loro attenzione	0	1	2	3	4	5	6
4. Cerco di tenermi occupato per evitare pensieri o emozioni	0	1	2	3	4	5	6
5. Agisco in modo coerente a come desidero vivere la mia vita	0	1	2	3	4	5	6
6. Sono talmente preso dai miei pensieri da non riuscire a fare le cose che più voglio fare	0	1	2	3	4	5	6
7. Faccio scelte in base a quello che per me è importante, anche quando mi costa fatica	0	1	2	3	4	5	6
8. Dico a me stesso che non dovrei avere certi pensieri	0	1	2	3	4	5	6
9. Faccio fatica a rimanere concentrato su ciò che accade nel presente	0	1	2	3	4	5	6
10. Mi comporto secondo i miei valori personali	0	1	2	3	4	5	6
11. Faccio di tutto per evitare situazioni che potrebbero suscitare pensieri, emozioni o sensazioni spiacevoli	0	1	2	3	4	5	6
12. Anche quando faccio cose che per me sono importanti, mi accorgo di farle senza prestare attenzione	0	1	2	3	4	5	6
13. Sono disposto a vivere pienamente qualsiasi pensiero, emozione e sensazione mi si presenti senza cercare di cambiarli o difendermene	0	1	2	3	4	5	6
14. Mi impegno a fare cose che per me sono importanti, anche quando lo trovo difficile	0	1	2	3	4	5	6
15. Faccio di tutto per tenere lontano le emozioni che mi fanno star male	0	1	2	3	4	5	6
16. Eseguo compiti o mansioni in modo automatico, senza rendermi conto di quello che sto facendo	0	1	2	3	4	5	6
17. Sono in grado di perseguire i miei piani a lungo termine anche quando procedono lentamente	0	1	2	3	4	5	6
18. Anche quando una cosa è importante per me, difficilmente la faccio se c'è il rischio che mi faccia star male	0	1	2	3	4	5	6
19. Mi sembra di fare le cose con il "pilota automatico", senza rendermi bene conto di quello che sto facendo	0	1	2	3	4	5	6
20. I pensieri sono solo pensieri: non controllano quello che faccio	0	1	2	3	4	5	6
21. Il mio comportamento rispecchia davvero i miei valori	0	1	2	3	4	5	6
22. Sono in grado di accogliere pensieri e emozioni così come vengono, senza cercare di controllarli o evitarli	0	1	2	3	4	5	6
23. Sono in grado di andare avanti quando qualcosa è importante per me	0	1	2	3	4	5	6

German CompACT

ID Code:	Datum:

Bitte bewerten Sie die folgenden 23 Aussagen anhand der folgenden Skala

0	1	2	3	4	5	6
Starke Ablehnung	Mäßige Ablehnung	Geringe Ablehnung	Weder Zustimmung noch Ablehnung	Geringe Zustimmung	Mäßige Zustimmung	Starke Zustimmung

1. Ich kann die Dinge benennen, die mir im Leben wirklich wichtig sind, und diese auch verfolgen	0	1	2	3	4	5	6
2. Eines meiner großen Ziele ist es, keine schmerzhaften Gefühle zu haben	0	1	2	3	4	5	6
3. Ich haste durch bedeutsame Aktivitäten, ohne sie wirklich bewusst zu erleben	0	1	2	3	4	5	6
4. Ich versuche, stets beschäftigt zu sein, um keine Gedanken oder Gefühle aufkommen zu lassen	0	1	2	3	4	5	6
5. Ich lebe so, wie ich mein Leben leben möchte.	0	1	2	3	4	5	6
6. Ich bin so in meinen Gedanken gefangen, dass es mich davon abhält, die Dinge zu tun, die ich am liebsten tun möchte	0	1	2	3	4	5	6
7. Ich treffe Entscheidungen danach, was mir wichtig ist, auch wenn es mich belastet	0	1	2	3	4	5	6
8. Ich sage mir, dass ich bestimmte Gedanken nicht haben sollte	0	1	2	3	4	5	6
9. Es fällt mir schwer, mich auf das zu konzentrieren, was gerade geschieht	0	1	2	3	4	5	6
10. Ich handle im Einklang mit meinen persönlichen Werten	0	1	2	3	4	5	6
11. Ich versuche alles, um Situationen zu vermeiden, die schwierige Gedanken, Gefühle oder Empfindungen hervorrufen könnten	0	1	2	3	4	5	6
12. Selbst wenn ich Dinge tue, die mir wichtig sind, merke ich, dass ich nicht bei der Sache bin	0	1	2	3	4	5	6
13. Ich bin bereit, alle aufkommenden Gedanken, Gefühle und Empfindungen voll zuzulassen, ohne zu versuchen, sie zu ändern oder abzuwehren	0	1	2	3	4	5	6
14. Ich nehme Dinge in Angriff, die für mich bedeutsam sind, auch wenn es mir schwer fällt	0	1	2	3	4	5	6
15. Ich bemühe mich sehr, beunruhigende Gefühle fernzuhalten	0	1	2	3	4	5	6
16. Ich erledige Aufgaben und Tätigkeiten automatisch, ohne mir recht bewusst zu sein, was ich gerade tue	0	1	2	3	4	5	6
17. Ich bin in der Lage, meine langfristigen Pläne zu verfolgen, auch wenn es langsam voran geht	0	1	2	3	4	5	6
18. Selbst wenn mir etwas wichtig ist, tue ich es eher nicht, wenn die Möglichkeit besteht, dass es mich beunruhigen könnte	0	1	2	3	4	5	6
19. Es kommt mir so vor, als wäre ich "auf Automatik" geschaltet, ohne dass ich bewusst wahrnehme, was ich tue	0	1	2	3	4	5	6
20. Gedanken sind nur Gedanken - sie bestimmen nicht, was ich tue	0	1	2	3	4	5	6
21. Meine Werte spiegeln sich wirklich in meinem Verhalten wider	0	1	2	3	4	5	6
22. Ich kann Gedanken und Gefühle so nehmen, wie sie kommen, ohne zu versuchen, sie zu kontrollieren oder zu vermeiden	0	1	2	3	4	5	6
23. Ich kann an etwas dranbleiben, wenn es mir wichtig ist	0	1	2	3	4	5	6

Spanish CompACT

CompACT

Código ID:	Fecha:

Por favor, evalúe las 23 frases siguientes mediante la escala que figura a continuación:

**0**	**1**	**2**	**3**	**4**	**5**	**6**
Muy en desacuerdo	Bastante en desacuerdo	Algo en desacuerdo	Ni de acuerdo ni en desacuerdo	Algo de acuerdo	Bastante de acuerdo	Muy de acuerdo

1. Soy capaz de decir qué cosas me importan de verdad en la vida y dedicarme a ellas.	0	1	2	3	4	5	6
2. Uno de mis grandes objetivos es vivir sin emociones dolorosas.	0	1	2	3	4	5	6
3. Hago a toda prisa actividades importantes para mí sin prestarles verdadera atención.	0	1	2	3	4	5	6
4. Intento mantenerme ocupado/a para evitar que me vengan pensamientos o sentimientos.	0	1	2	3	4	5	6
5. Mis maneras de actuar concuerdan con la forma en que deseo vivir la vida.	0	1	2	3	4	5	6
6. Me quedo tan absorto/a en mis pensamientos que soy incapaz de hacer las cosas que más deseo.	0	1	2	3	4	5	6
7. Elijo hacer lo que es importante para mí, aunque resulte estresante.	0	1	2	3	4	5	6
8. Me digo a mí mismo/a que no debería tener ciertos pensamientos.	0	1	2	3	4	5	6
9. Me resulta difícil mantenerme centrado/a en lo que sucede en el presente.	0	1	2	3	4	5	6
10. Me comporto de forma fiel a mis valores personales.	0	1	2	3	4	5	6
11. Me esfuerzo por evitar situaciones que puedan traerme pensamientos, sentimientos o sensaciones difíciles.	0	1	2	3	4	5	6
12. Incluso cuando estoy haciendo las cosas que me importan, veo que las estoy haciendo sin prestar atención.	0	1	2	3	4	5	6
13. Estoy dispuesto/a a sentir plenamente todos los pensamientos, sentimientos y sensaciones que me surjan, sin intentar cambiarlos ni pelear contra ellos.	0	1	2	3	4	5	6
14. Llevo a cabo cosas que son importantes para mí incluso cuando me resulta difícil hacerlo.	0	1	2	3	4	5	6
15. Me esfuerzo mucho por apartar los sentimientos que me puedan alterar.	0	1	2	3	4	5	6
16. Hago trabajos o tareas mecánicamente, sin darme cuenta de lo que estoy haciendo.	0	1	2	3	4	5	6
17. Soy capaz de seguir mis planes a largo plazo incluso cuando avanzo despacio.	0	1	2	3	4	5	6
18. Aunque algo me resulte importante, rara vez lo hago si existe la posibilidad de que me altere.	0	1	2	3	4	5	6
19. Parece que «voy con el piloto automático» sin fijarme mucho en lo que estoy haciendo.	0	1	2	3	4	5	6
20. Los pensamientos son solo pensamientos y no controlan lo que hago.	0	1	2	3	4	5	6
21. Mis valores se reflejan fielmente en mi comportamiento.	0	1	2	3	4	5	6
22. Soy capaz de aceptar los pensamientos y sentimientos tal como llegan, sin intentar controlarlos ni evitarlos.	0	1	2	3	4	5	6
23. Soy capaz de seguir adelante con las cosas cuando me importan.	0	1	2	3	4	5	6
